# Inhibition of GSK-3 Ameliorates Aβ Pathology in an Adult-Onset *Drosophila* Model of Alzheimer's Disease

**DOI:** 10.1371/journal.pgen.1001087

**Published:** 2010-09-02

**Authors:** Oyinkan Sofola, Fiona Kerr, Iain Rogers, Richard Killick, Hrvoje Augustin, Carina Gandy, Marcus J. Allen, John Hardy, Simon Lovestone, Linda Partridge

**Affiliations:** 1 Institute of Healthy Ageing and Research Department of Genetics, Evolution, and Environment, University College London, London, United Kingdom; 2 Max Planck Institute for Biology of Ageing, Köln, Germany; 3 Institute of Psychiatry, King's College London, London, United Kingdom; 4 School of Biosciences, University of Kent, Canterbury, United Kingdom; 5 Institute of Neurology, University College London, London, United Kingdom; Stanford University School of Medicine, United States of America

## Abstract

Aβ peptide accumulation is thought to be the primary event in the pathogenesis of Alzheimer's disease (AD), with downstream neurotoxic effects including the hyperphosphorylation of tau protein. Glycogen synthase kinase-3 (GSK-3) is increasingly implicated as playing a pivotal role in this amyloid cascade. We have developed an adult-onset *Drosophila* model of AD, using an inducible gene expression system to express Arctic mutant Aβ42 specifically in adult neurons, to avoid developmental effects. Aβ42 accumulated with age in these flies and they displayed increased mortality together with progressive neuronal dysfunction, but in the apparent absence of neuronal loss. This fly model can thus be used to examine the role of events during adulthood and early AD aetiology. Expression of Aβ42 in adult neurons increased GSK-3 activity, and inhibition of GSK-3 (either genetically or pharmacologically by lithium treatment) rescued Aβ42 toxicity. Aβ42 pathogenesis was also reduced by removal of endogenous fly tau; but, within the limits of detection of available methods, tau phosphorylation did not appear to be altered in flies expressing Aβ42. The GSK-3–mediated effects on Aβ42 toxicity appear to be at least in part mediated by tau-independent mechanisms, because the protective effect of lithium alone was greater than that of the removal of tau alone. Finally, Aβ42 levels were reduced upon GSK-3 inhibition, pointing to a direct role of GSK-3 in the regulation of Aβ42 peptide level, in the absence of APP processing. Our study points to the need both to identify the mechanisms by which GSK-3 modulates Aβ42 levels in the fly and to determine if similar mechanisms are present in mammals, and it supports the potential therapeutic use of GSK-3 inhibitors in AD.

## Author Summary

Alzheimer's disease (AD) is the leading cause of dementia in the ageing population. Symptoms include memory loss and decline in understanding and reasoning. Alois Alzheimer, who reported the first case of AD, observed plaques and tangles in the brains of patients. The plaques are made up of amyloid protein, while the tangles are of tau protein. One of the main scientific ideas about AD is that it starts with build-up of amyloid, which then alters tau protein, causing the disease. Another protein, called GSK-3, also seems to play a part. Simple invertebrates such as flies are useful for understanding human diseases. We have created an AD model in the fruit fly *Drosophila* where amyloid protein is present in the nerve cells of the adult fly; this caused the flies to be impaired in their survival, nerve function, and behavior. We found that amyloid increased the activity of GSK-3, and so we experimentally turned down its activity and found that this improved the survival and behavior of the flies. Importantly, turning down the activity of GSK-3 in flies that did not have amyloid did not seem to harm them. GSK-3 could therefore be a good target for drugs against AD.

## Introduction

Alzheimer's disease (AD) is the leading cause of dementia in the ageing population. Symptoms include, but are not limited to, memory loss, cognitive decline, and deterioration of language skills. The pathological hallmarks of AD are the presence of plaques and neurofibrillary tangles [1]. The tangles are composed of hyperphosphorylated tau protein while the plaques are comprised of amyloid beta (Aβ) peptides, various species of which are derived from the amyloid precursor protein (APP), the most abundant being Aβ40 and Aβ42 [2]. AD-causing mutations either increase the level of Aβ42 or the ratio of Aβ42/Aβ40, indicating that this is the more toxic form of the peptide [2].

The leading candidate explanation for the molecular basis of AD pathology is the amyloid cascade hypothesis. This states that the Aβ protein initiates the disease process, activating downstream neurotoxic mechanisms including the dysregulation of tau. Perhaps the strongest support for the amyloid cascade hypothesis is that all of the mutations implicated in early-onset, familial AD, such as the Aβ Arctic mutation, increase the aggregation or production of Aβ [1]. Although tau mutations exist, none have been linked to familial AD, but rather to fronto-temporal dementia, in which Aβ plaques are absent [3], [4]. The amyloid cascade has also been tested experimentally in various ways. For example, a double transgenic mouse model expressing APP-V7171 and Tau-P301L, develops amyloid pathology similarly to mice transgenic for APP-V7171 alone, whereas tauopathy is dramatically enhanced in the double transgenic compared to mice transgenic for Tau-P301L alone. This implies that Aβ pathology affects tauopathy but not *vice versa* [5]. Also, clearance of Aβ using Aβ-specific antibodies reduced early tau burden, while elevating tau burden in transgenic mice had no effect on Aβ accumulation [6], [7]. Furthermore, a reduction in tau levels rescued learning and memory impairment induced by Aβ in a mouse model expressing human APP [8].

Aβ increases the phosphorylation of tau protein and concomitantly activates glycogen synthase kinase, GSK-3 [9], [10]. GSK-3 is a multi-functional kinase involved in regulating various cellular processes, including growth and differentiation [9], [11]. There are two isoforms of the protein, GSK-3α and GSK-3β. They share 98% identity within their kinase domain, but are not functionally identical, although both have been suggested to be involved in AD pathogenesis [11]. GSK-3α has been implicated in the amyloidogenic processing of APP to yield Aβ peptides [12], while GSK-3β has been implicated in the tau-related pathogenesis of AD, by colocalizing with tau tangles and phosphorylating tau [9]. As yet, the exact role of GSK-3 in the generation of Aβ peptides is not known. GSK-3 itself is also regulated by phosphorylation. Phosphorylation at Ser9 of GSK-3β and the equivalent Ser21 of GSK-3α inhibits activity, while phosphorylation at Tyr216/Tyr279 of GSK-3β and GSK-3α, respectively, is thought to increase activity [13].

Remarkable similarities are seen between double transgenic mice expressing tau either with APP or with GSK-3β. This finding is consistent with the hypothesis that amyloid acts via activation of GSK-3 to modulate tau function [5], [9], [14]. Lithium chloride, which is used as a mood stabilizing agent in patients with bipolar disorders, inhibits GSK-3 activity, either by competing with magnesium ions [15] or by increasing Ser9 phosphorylation [16]. Lithium reduces amyloid production by altering the role of GSK-3α in APP processing/cleavage; selective inhibition of GSK-3 by siRNA or expressing a dominant negative form of GSK-3 also decreases Aβ production in cultured cells and mice [12], [17]. Furthermore, lithium reduces both tau phosphorylation at several GSK-3 epitopes and tauopathy in a mouse model expressing mutant human tau [12], [18]. However, in another study, lithium was seen to reduce tau phosphorylation but not to affect Aβ load in a triple mutant mouse expressing human APP_swe_, human tau_P301L_ and with mutant presenilin 1 PS1_M146V_ knock-in. The differing observations might be due to variations in the age at which the mice were treated with lithium, as suggested by the authors [19].

Fruit flies, *Drosophila melanogaster*, can provide useful invertebrate models of neurodegeneration because of their complex brains, short lifespans and relative ease of genetic manipulation. Several fly models of aspects of AD biology have been made, including ones that over-express either *Drosophila* or human tau, and show neuronal dysfunction phenotypes [20]–[22]. Co-expression of human tau protein with Shaggy (Sgg), the *Drosophila* homologue of GSK-3 [23], exacerbates these neurotoxic phenotypes and leads to the appearance of neurofibrillary tangles [22], [24], [25]. Fly models expressing Aβ peptides have also been generated, and show neurodegeneration and amyloid deposits [26], [28]. Although an APP orthologue exists in flies, the Aβ sequence is not conserved, and *Drosophila* models directly expressing Aβ allow study of Aβ toxicity in the absence of any endogenous amyloid production [29].

In this study we have generated a fly model that expresses Arctic mutant Aβ42 peptide in the nervous system of adult flies, using an inducible system for gene expression, because we wished to understand the underlying mechanism of disease progression of AD in adults, without complications from developmental effects. We first characterised this model, and then used it to investigate the amyloid cascade hypothesis, by modulating the levels of endogenous fly tau and examining the effects on phenotypes consequent upon expression of Aβ. We also investigated the requirement for GSK-3 in Aβ pathology and its role in direct regulation of Aβ peptides.

## Results

### Arctic Aβ42 expression can be induced in adult *Drosophila* neurons

**Figure 1:**
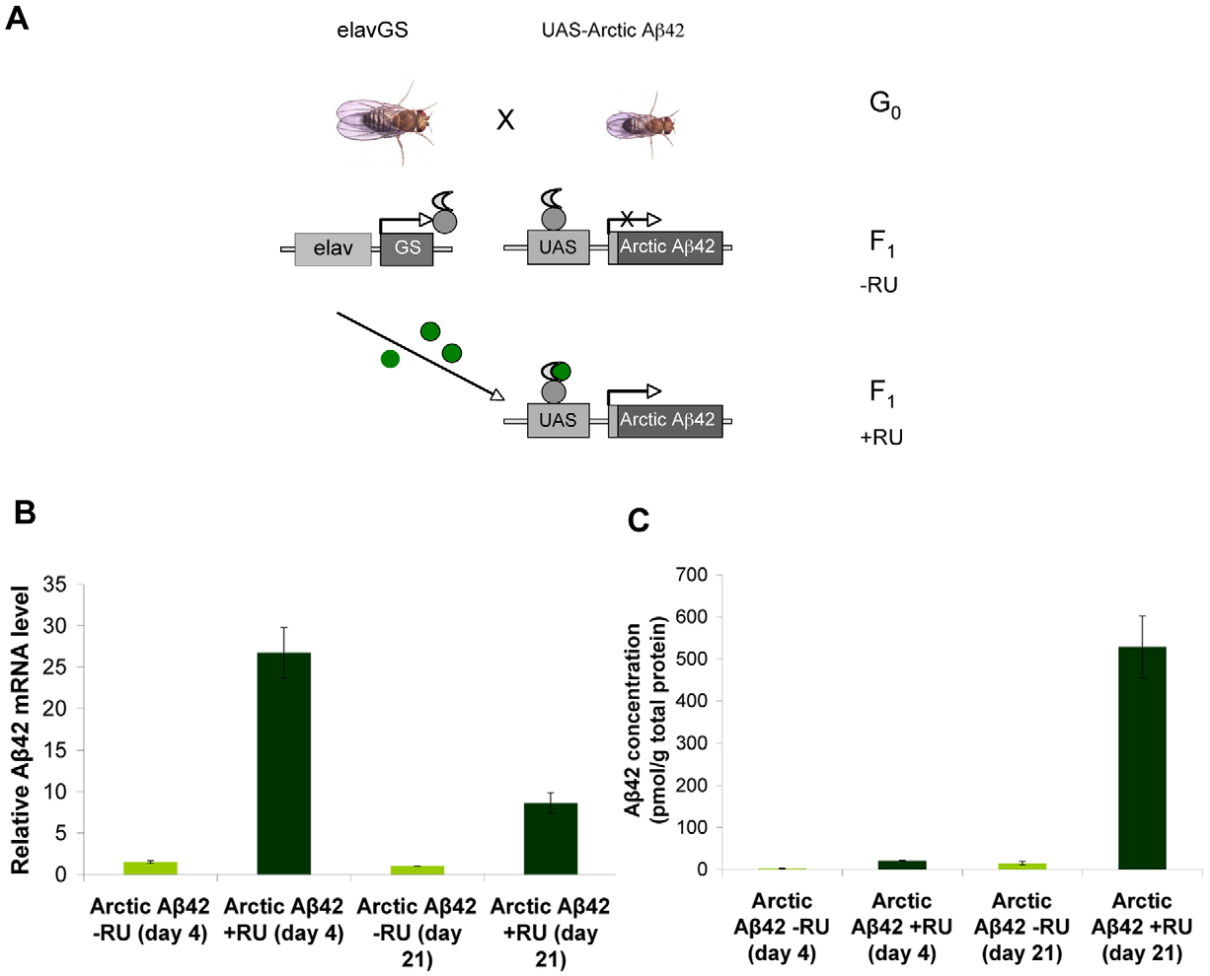
Adult-onset induction of Arctic Aβ42 peptide in the *Drosophila* nervous system. (A) A schematic representation of the GeneSwitch-UAS expression system (based on [30]). Driver lines expressing the transcriptional activator GeneSwitch under control of the nervous system-specific elav promoter (elavGS) are crossed to flies expressing an Aβ transgene fused to a GAL4-binding upstream activation sequence (UAS-Aβ). In the absence of the activator mifepristone (RU486; −RU), the GeneSwitch protein is expressed in neurons but remains transcriptionally silent, so that Aβ is not expressed. Following treatment with RU486 (+RU; green) the GeneSwitch protein is transcriptionally activated, binds to UAS and thus mediates expression of Aβ peptide specifically in the fly nervous system. Aβ42 RNA (B) and protein (C) levels were quantified at four days and 21 days post-RU486 treatment (see Materials and Methods). Data are presented as means ± SEM and were analysed by two-way ANOVA and Tukey's honestly significant difference (HSD) post-hoc comparisons. *P*<0.05 comparing Aβ RNA expression in RU486-treated UAS-ArcAβ42/+;elavGS/+ flies to their −RU486 controls at both time-points (Tukey's HSD). *P*<0.01 comparing Aβ42 protein levels in RU486-treated UAS-ArcAβ42/+;elavGS/+ flies to untreated controls at both time-points.

To generate an adult-onset fly model of Alzheimer's disease, we expressed Arctic mutant Aβ42 peptides using an inducible pan-neuronal driver. An elav GeneSwitch (elavGS) driver line [30]–[31] that has been used previously to develop an adult-onset *Drosophila* model of spinocerebellar ataxia (SCA) [32] was used to direct expression of a UAS-Arctic Aβ42 transgene [33] both spatially and temporally, to neurons of the adult fly (Figure 1A). A UAS-Aβ40 line [33] was used as a control for over-expression of non-toxic forms of Aβ in fly neurons, since this form of the peptide has previously been shown to have no detrimental effect in flies [26]–[28].

We measured expression of Aβ peptides in adult neurons when we treated elavGS;UAS-Arctic Aβ42 flies with the activator mifepristone (RU486;RU) from two days post-eclosion, by measuring RNA and protein levels at 4 and 21 days into treatment (Figure 1B and 1C). Aβ transcripts were clearly elevated in RU-treated elavGS;UAS-Arctic Aβ42 flies in comparison with untreated (−RU) flies at both time-points (Figure 1B). Moreover, an Aβ42-specific ELISA confirmed that Aβ42 protein was elevated in elavGS;UAS-Arctic Aβ42 (+RU) flies compared to untreated (−RU) flies and that the level of protein increased with age (Figure 1C). Since RNA transcript level decreased with age, this age-dependent accumulation of Aβ42 protein is most likely to be attributable to an increased rate of translation of the protein relative to the rate of protein degradation.

**Figure 2:**
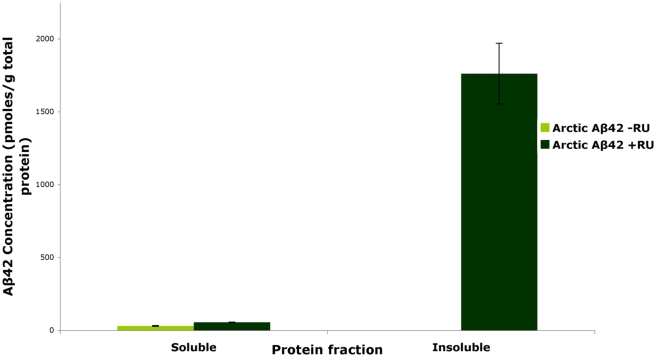
Arctic Aβ42 peptide in the adult *Drosophila* nervous system is mostly in an insoluble fibrillar state. In the absence of the activator mifepristone (RU486; −RU), a negligible amount of soluble protein is observed at day 15. Following treatment with RU486 (+RU; dark green) the Aβ peptide expression is seen in both soluble and insoluble fractions with a significant proportion observed in the insoluble fraction. Data are presented as means ± SEM and were analysed by ANOVA, *P*<0.01 when protein levels of soluble and insoluble fractions of Aβ42 expressing flies were compared.

Aggregation of Aβ has been shown to be of critical importance for its pathogenicity [34]. Therefore, we assessed the state of aggregation of Aβ42 in the mutant flies by separating soluble and insoluble protein fractions from fly brain extracts. At day 15, when the first signs of pathology were observed in the Arctic Aβ42 flies (see below), we found that most of the Aβ42 protein had accumulated into an insoluble, fibrillar form (Figure 2), consistent with the aggregation-promoting effects of the Arctic mutation [35].

Overall these results confirm that the elavGS-UAS system used in this study is sufficient to induce over-expression of Aβ peptides specifically in the adult fly nervous system, and that Arctic mutant Aβ42 protein accumulates with age.

### Over-expression of Arctic Aβ42 peptide in adult neurons increases mortality and induces neuronal dysfunction in *Drosophila*, without evidence of neuronal cell loss

Previously published studies have shown that constitutive expression of Arctic Aβ42 peptide in fly neurons significantly shortens lifespan, induces behavioural impairments and causes neuronal death [26]–[28]. To determine whether adult-onset expression of Arctic Aβ42 peptide in neurons causes similar phenotypes, we examined survival, neuronal and behavioural dysfunction in our inducible *Drosophila* model of AD.

**Figure 3:**
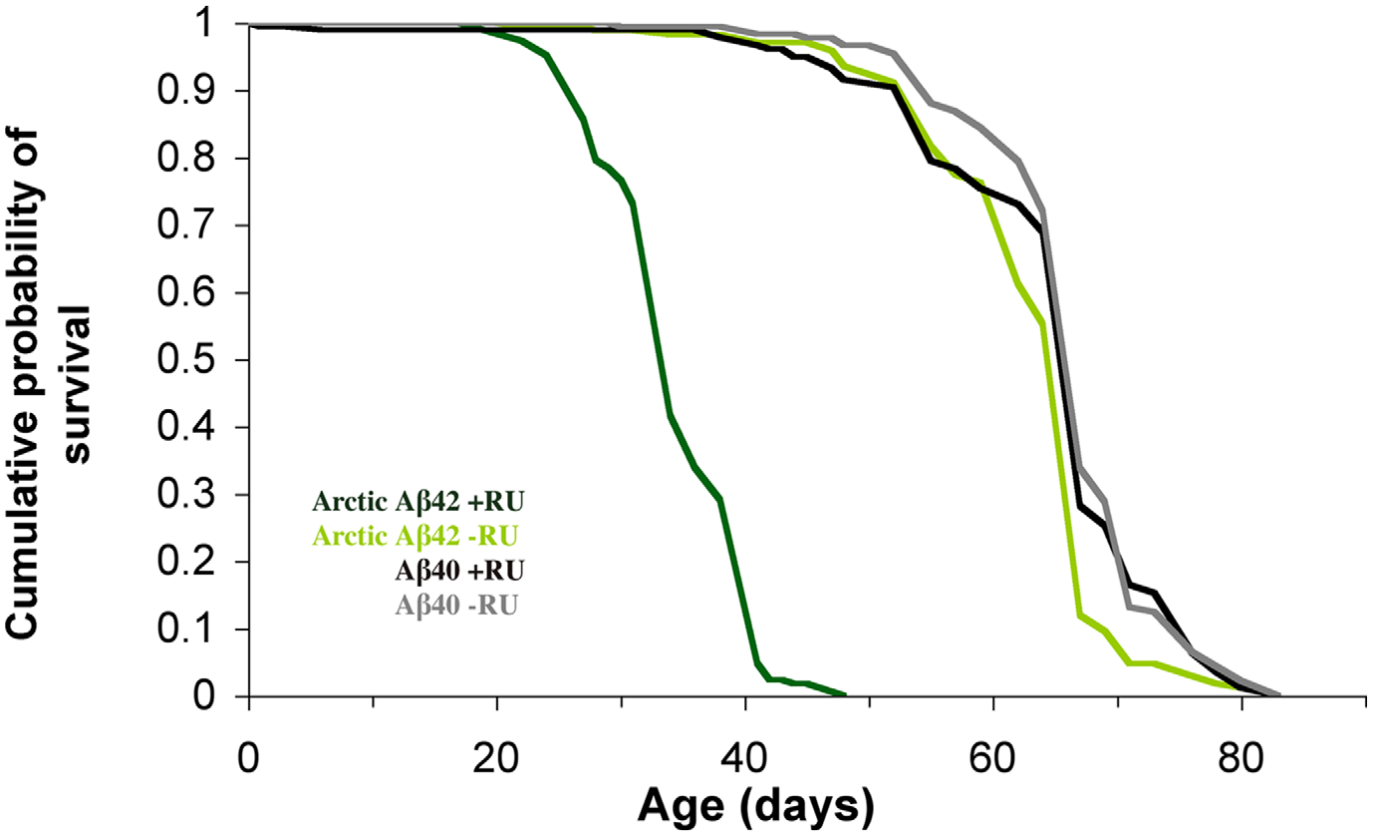
Expression of Arctic Aβ42 specifically in the adult nervous system shortens lifespan. Lifespans were determined as described in materials & methods. Survival curves are depicted and data were compared using the log-rank test. *P*<0.01 comparing median lifespan of UAS-ArcAβ42/+;elavGS/+ +RU flies to their −RU controls. *P*<0.01 comparing UAS-ArcAβ42/+;elavGS/+ +RU flies to UAS-Aβ40/+;elavGS/+ +RU controls. Induction of Aβ40 in the adult nervous system did not alter lifespan in comparison to non-RU486-treated controls.

First, we measured the effects of Arctic Aβ42 expression on lifespan, by treating elavGS;UAS-Arctic Aβ42 and elavGS;UAS-Aβ40 flies with RU from two days post-eclosion and recording their survival. Expression of Arctic Aβ42 in adult neurons shortened median lifespan by about 50% and maximum lifespan by about 45% in comparison to non-RU-treated flies, and to Aβ40 +RU and −RU control flies (Figure 3), demonstrating a specific lifespan-shortening effect of Arctic Aβ42 compared to the Aβ40 form of the peptide.

**Figure 4:**
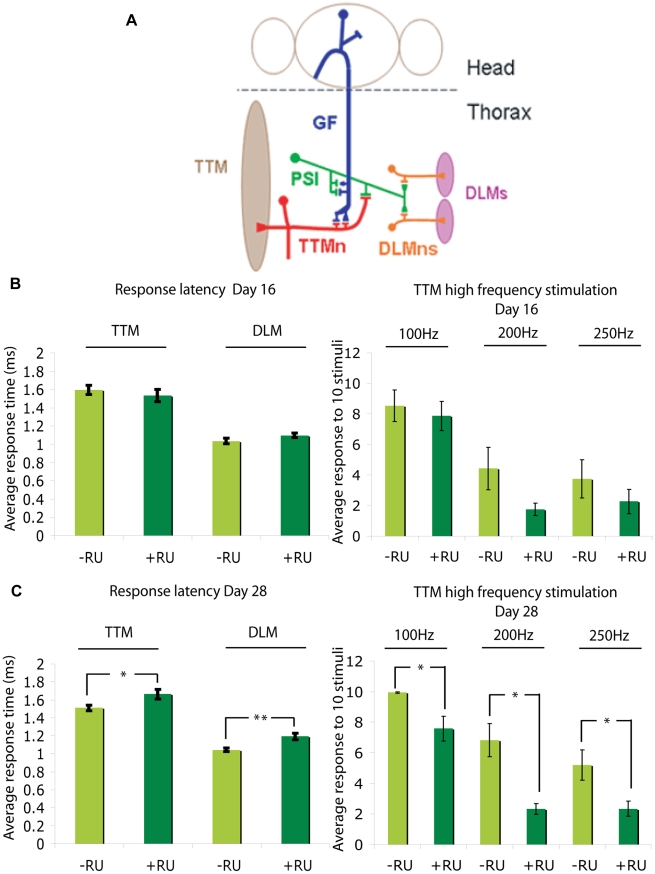
Arctic Aβ42 peptides induce progressive, adult-onset neuronal defects in *Drosophila*. (A) A schematic illustration of the *Drosophila* giant fibre system (GFS; adapted from [74]). Giant fibres (GFs; blue) relay signals from the brain to the thoracic musculature via mixed electrochemical synapses with the motorneurons (TTMn, red) of the tergotrancheral muscle (TTM; left), and the peripherally synapsing interneuron (PSI; green), which subsequently forms chemical synapses with the motorneurons (DLMn; orange) of the dorsal longitudinal muscles (DLM; right). Note only one of the TTMn axons is shown exiting the nervous system and contacting the muscle on the left hand side and one set of the DLMns and corresponding neuromuscular junctions are depicted on the right hand side. GFS activity was measured in UAS-ArcAβ42;elavGS flies at (B) 16 days and (C) 28 days post-RU486 treatment (see Materials and Methods); parameters measured were the latencies from GF stimulation to muscle response (response latency DLM and TTM) and the stability of the response to high frequency stimulation at 100, 200 and 250 Hz (high frequency stimulation TTM). Data are presented as the mean response ± SEM and were analysed by student's t-test, at each time point, on log-derived data. *P<0.05, **P<0.01 comparing response latency or response to high frequency stimulation of UAS-ArcAβ42/+;elavGS/+ +RU flies to −RU controls at 28 days post-induction.

Next, we determined whether adult-onset expression of Arctic Aβ42 peptide in fly neurons caused neuronal toxicity, by analysing neuronal function. As a direct measure of physiological activity, we examined the electrophysiological responses of the *Drosophila* giant fibre system (GFS; Figure 4A). Adult elavGS;UAS-Arctic Aβ42 flies were fed + or − RU486 media from two days post-eclosion, and GFS activity measured at day 16 and day 28 into treatment. Giant fibres (GF) were stimulated via electrodes inserted inside the compound eye, and post-synaptic potentials recorded in the tergotrochanteral muscle (TTM) and the dorsal longitudinal flight muscle (DLM) (Figure 4A); parameters measured were the latency from GF stimulation to muscle response and the stability of the response to high frequency stimulation. At day 16, response latencies in the TTM, DLM and the TTM to high frequency stimulation were comparable between elavGS;UAS-Arctic Aβ42 flies on + and − RU486 food (Figure 4B and Figure S1). However, at day 28, expression of Arctic Aβ42 peptide significantly increased the response latency measured in both the TTM and DLM, and inhibited the stability of the TTM response to high frequency stimulation (at 100, 200 and 250 Hz) in comparison to untreated control flies (Figure 4C and Figure S1). This indicates a progressive neuronal dysfunction following adult-onset induction of Arctic Aβ42, with young flies exhibiting no dysfunction in the GFS, while older flies showed obvious defects in response to both a single stimulus and to high frequency stimuli.

**Figure 5:**
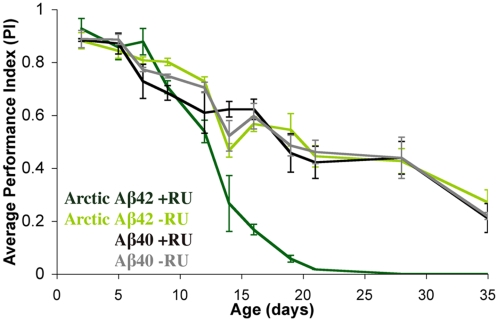
Expression of Arctic Aβ42 peptides in the adult fly nervous system causes locomotor dysfunction. Climbing ability of UAS-ArcAβ42/+;elavGS/+ and UAS-Aβ40/+;elavGS/+ flies on + and − RU486 SY medium was assessed at the indicated time-points (see Materials and Methods). Data are presented as the average performance index (PI) ± SEM and were compared using two-way ANOVA and Tukey's honestly significant difference (HSD) post-hoc analyses (number of independent tests (n) = 3, number of flies per group (n_t_) = 39–45). *P<0.05 comparing PI of UAS-ArcAβ42/+;elavGS/+ +RU486 flies to that of untreated and Aβ40 over-expressing controls at the indicated time points (Tukey's HSD).

As a behavioural measure of neuronal dysfunction in our inducible model, locomotor activity was assessed using a negative geotaxis (climbing) assay that has been used extensively to characterise fly models of neurodegenerative diseases [33], [36]. *Drosophila* display an age-related decline in climbing behaviour, and this was apparent in the non-RU-treated and elavGS;UAS-Aβ40 +RU control flies used in the current study (Figure 5). We found that flies expressing Arctic Aβ42 displayed a reduced negative geotaxis in comparison to their −RU control flies and the Aβ40 +RU and −RU flies (Figure 5). The climbing behaviour of the Arctic Aβ42 flies had declined to a level by day 15 that was reached by the control flies only by day 28.

Finally, we quantified neuronal loss, as measured by the number of cell bodies in one hemisphere, in flies over-expressing Arctic Aβ42 peptide in adult neurons compared to non-expressing controls. No neuronal loss was evident in the brains of these flies (Figure S2).

Collectively, these data demonstrate that expression of Arctic Aβ42 specifically in the neurons of the adult fly leads to early death and progressive neuronal dysfunction, in the apparent absence of neuronal loss. Hence we have successfully developed an inducible *Drosophila* model of AD that will provide a useful system in which to further investigate the potential mechanisms underlying pathogenesis in Alzheimer's disease, without any confounding effects on neuronal development.

### Adult-onset expression of Aβ42 increases the activity of Shaggy in the adult nervous system

**Figure 6:**
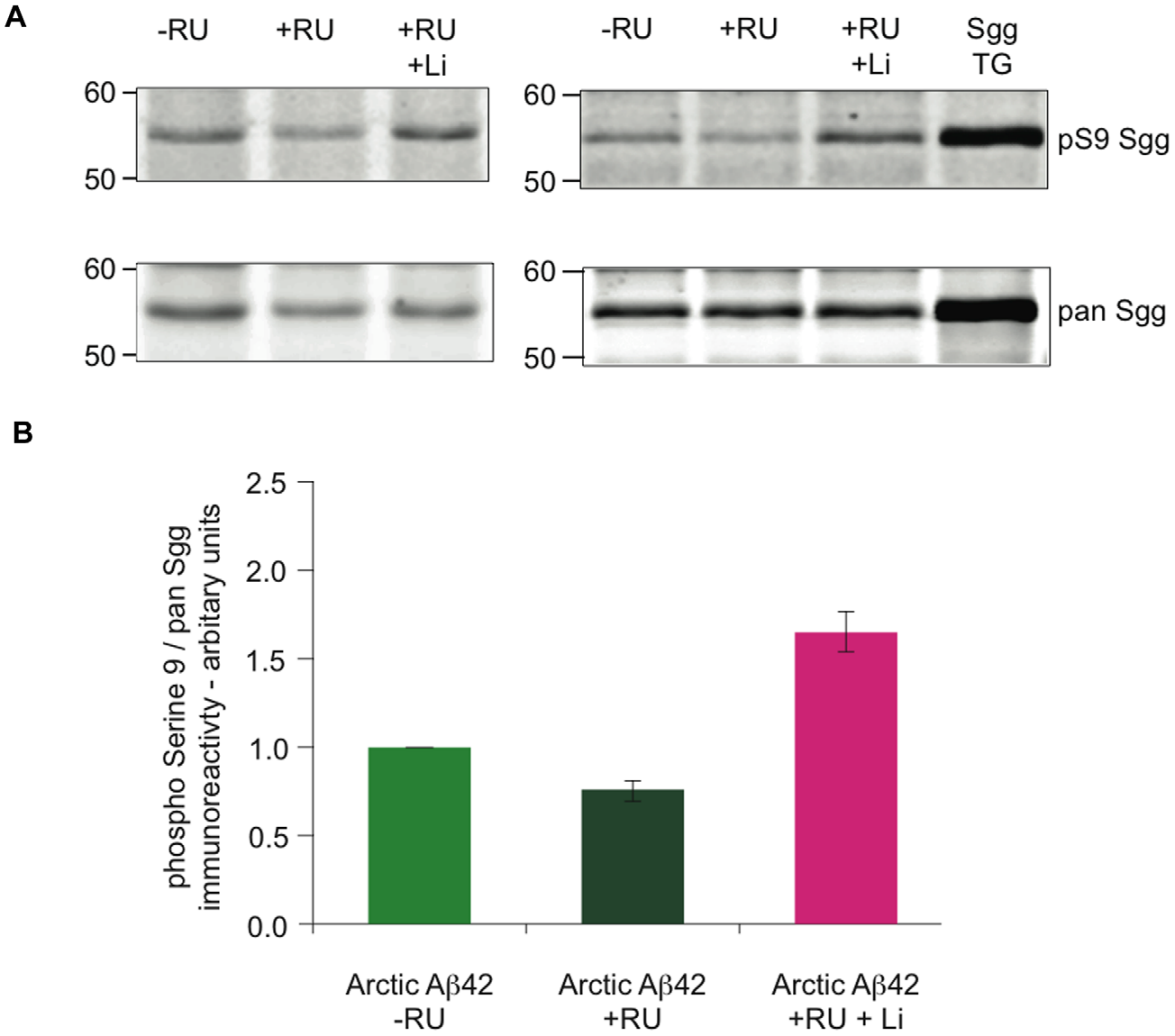
Flies expressing Aβ42 in adult neurons show a decrease in Shaggy inhibitory Ser9 phosphorylation. (A) Western blot analyses for pan-Sgg and phospho Ser9 Sgg revealed a decrease in Ser9 phosphorylation in flies expressing Aβ42 by RU induction for 15 days (UAS-ArcAβ42/+;elavGS/+ or UAS-ArcAβ42/GFP;elavGS/+) in comparison to their −RU controls, while an increase in Ser9 phosphorylation was observed when the Aβ42 expressing flies were fed lithium (+RU +Li). Flies over expressing Sgg were used as positive control. (B) Quantification of the western blot analysis in (A), n = 3, is depicted in the bar chart, with significant differences seen between ArcAβ42/+;elavGS/+ +RU flies to −RU controls (*P*<0.01), and between ArcAβ42/+;elavGS/+ +RU +Li flies to +RU controls (*P*<0.001).

Because of the described role of GSK-3 in Alzheimer's disease, we investigated the activity of the fly orthologue of GSK-3, Sgg, in the Aβ42-expressing flies. Phosphorylation at Ser9 of Sgg is important in suppressing its kinase activity. We found that expression of Arctic Aβ42 in the adult nervous system decreased the Ser9 phosphorylation level of Sgg, indicating an up-regulation of the activity of the kinase (see Figure 6). This increase in Sgg activity could have contributed to the toxicity we observed in our Aβ42 expressing flies. Lithium is a GSK-3 inhibitor, and we therefore tested its effect on Ser9 phosphorylation in the Aβ42 flies and, indeed, we found an increase in Ser9 phosphorylation compared to untreated controls (Figure 6).

### Inhibition of Shaggy activity in the adult nervous system suppresses the toxicity of Arctic Aβ42

**Figure 7:**
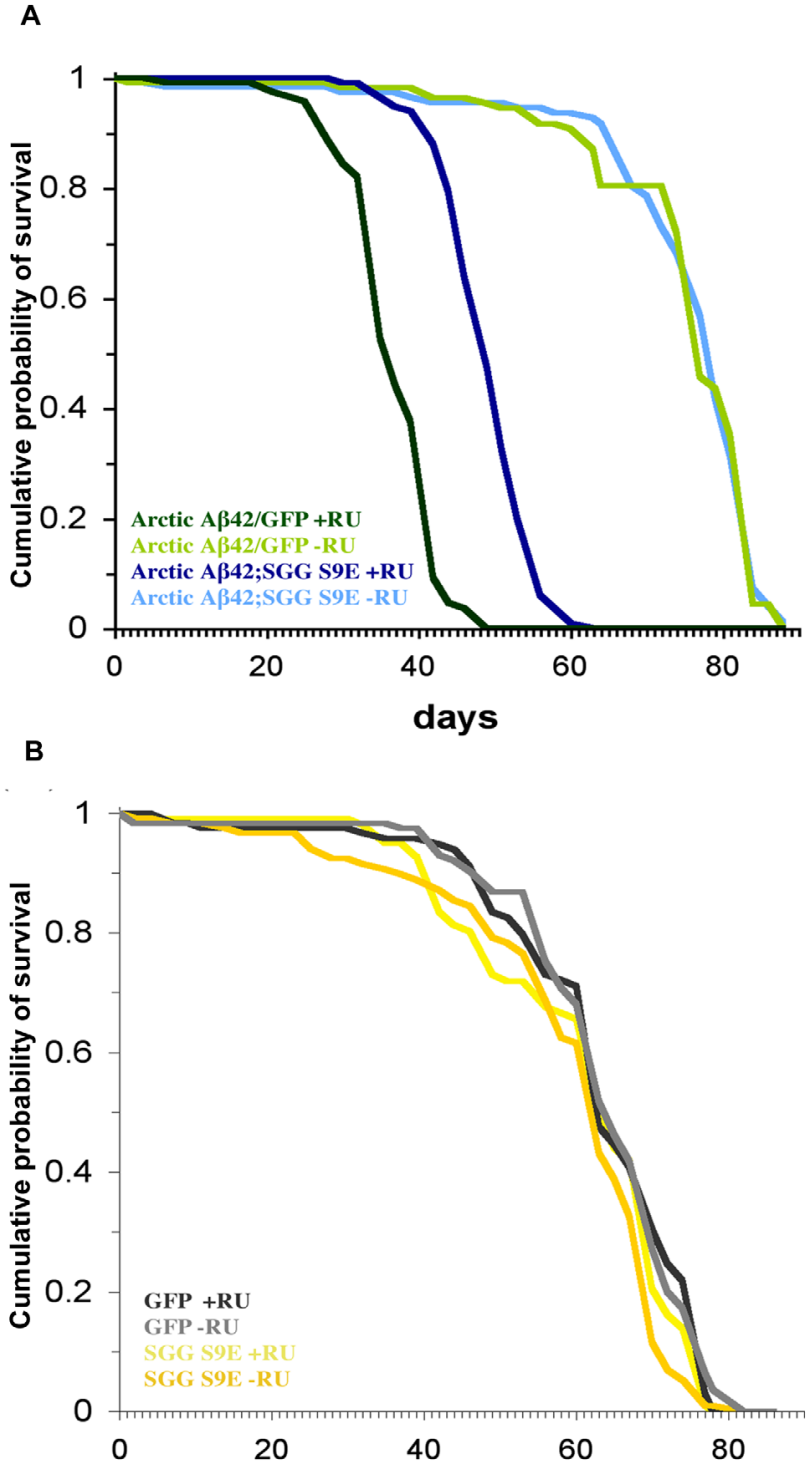
Expression of the dominant negative mutant Shaggy S9E in the adult nervous system extends the median and maximum lifespan of Arctic Aβ42 flies. (A). Survival curves of flies co-expressing Arctic Aβ42 and SggS9E are depicted and data were compared using the log-rank test. *P*<0.001 comparing UAS-ArcAβ42/+;elavGS/UAS-SggS9E +RU flies to UAS-ArcAβ42/UAS-gfp;elavGS/+ +RU controls. No significant difference was seen in the −RU controls. (B). Expression of Shaggy S9E alone did not affect control lifespan. No significant difference was observed comparing elavGS/UAS-SggS9E +RU to either elavGS/UAS-SggS9E −RU, or UAS-gfp/elavGS/+ +RU control lifespans.

**Figure 8:**
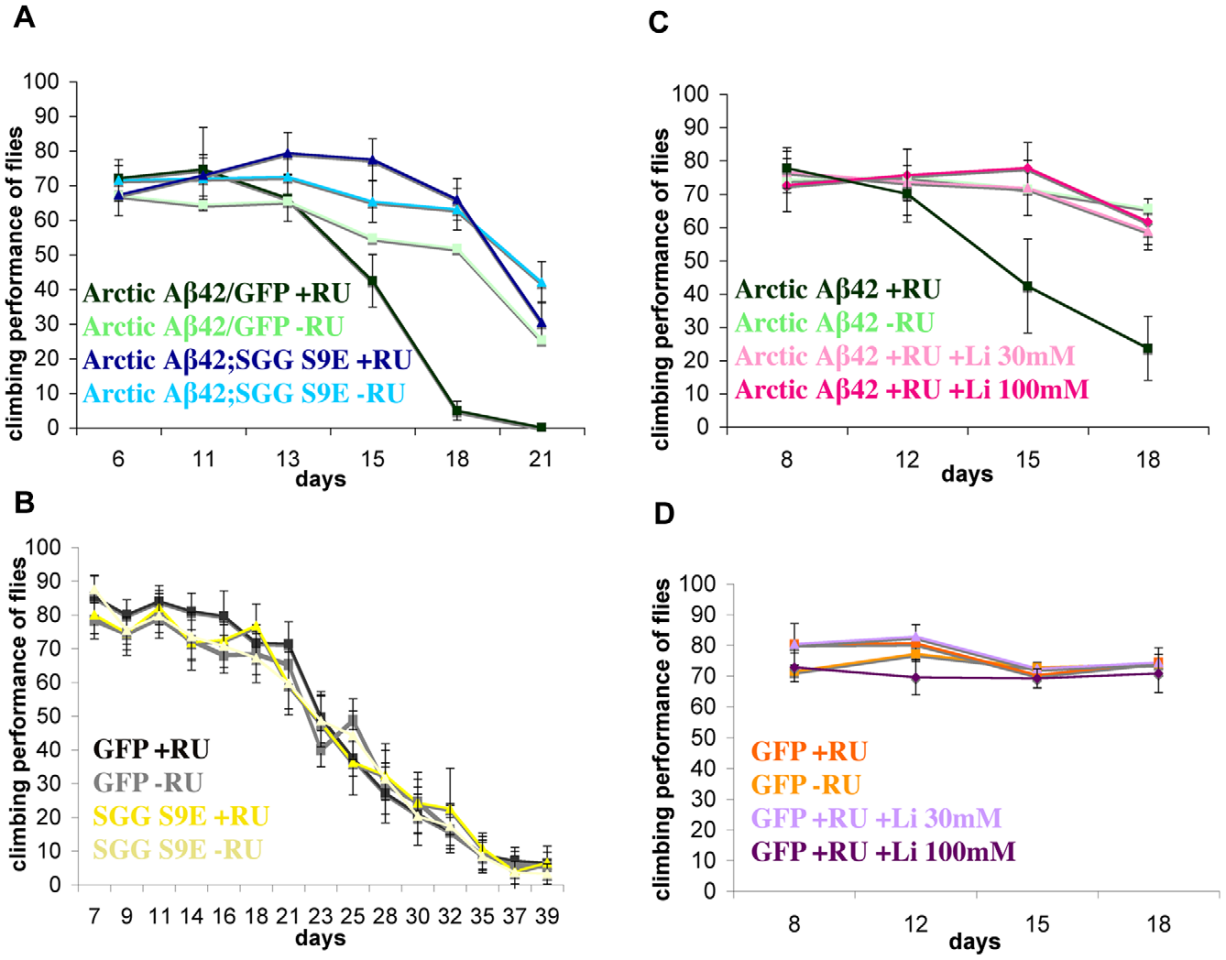
Inhibition of Shaggy, either by expression of SggS9E in the adult nervous system or treatment with lithium, suppresses the locomotor dysfunction phenotype of Arctic Aβ42 flies. (A) Climbing ability of UAS-ArcAβ42/UAS-gfp;elavGS/+ and UAS-ArcAβ42/+;elavGS/UAS-SggS9E flies on +RU486 SY medium was assessed at the indicated time-points (see Materials and Methods). Data are presented as the percentage climbing performance of flies ± SD. *P*<0.001 when UAS-ArcAβ42/UAS-gfp;elavGS/+ and UAS-ArcAβ42/+;elavGS/UAS-SggS9E flies are compared at day 21 (one-way ANOVA, number of independent tests (n) = 3). Graph shows one representative data of repeated experiments. (B) Expression of Shaggy S9E alone does not reduce climbing ability of control flies. elavGS/UAS-SggS9E +RU flies display a similar locomotor function compared to both elavGS/UAS-SggS9E −RU and UAS-gfp/+;elavGS/+ +RU control flies. (C) Climbing ability of UAS-ArcAβ42/+;elavGS/+ and UAS-ArcAβ42/UAS-gfp;elavGS/+ on +RU486 SY medium was assessed in the presence and absence of lithium chloride (30mM and 100mM). *P*<0.001 when UAS-ArcAβ42 flies were fed lithium and compared to flies not fed lithium at day 18 (one-way ANOVA, n = 3). Graph shows one representative data of repeated experiments. (D) Lithium had no effect on negative geotaxis of control flies. UAS-gfp/+;elavGS/+ +RU and UAS-gfp/+;elavGS/+ +RU in the presence of lithium had similar locomotor function. The crosses were performed at 27°C.

We next investigated if the increase in Sgg activity that we observed in the Artic Aβ42-expressing flies contributed to Aβ42 toxicity. To do this, we co-expressed in adult neurons a dominant negative form of Sgg, the S9E mutant, which mimics an inhibited state of the kinase [37], [38] and renders it inactive. Expression of this dominant-negative Sgg increased the median and maximum lifespan of flies expressing Arctic Aβ42. Flies co-expressing Arctic Aβ42 and the dominant negative mutant S9E lived significantly longer than control flies co-expressing Arctic Aβ42 and GFP (Figure 7), to control for any titration effect of GAL4 in the presence of a second UAS-transgene. Furthermore, inactivation of Sgg, either by expressing the dominant negative mutant S9E or feeding the flies lithium in adulthood, significantly suppressed the climbing deficit of the Arctic Aβ42-expressing flies (Figure 8). Two different doses of lithium (30mM and 100mM) both rescued the climbing deficit of the Aβ42-expressing flies. These data demonstrate that inhibiting the activity of Sgg in neurons in adults suppresses the adult onset Arctic Aβ42 induced toxicity, and demonstrate experimentally a functional role of GSK-3 in mediating Aβ42 toxicity.

### Arctic Aβ42 toxicity, and protection by Shaggy inhibition, is not mediated predominantly through alterations in tau phosphorylation

**Figure 9:**
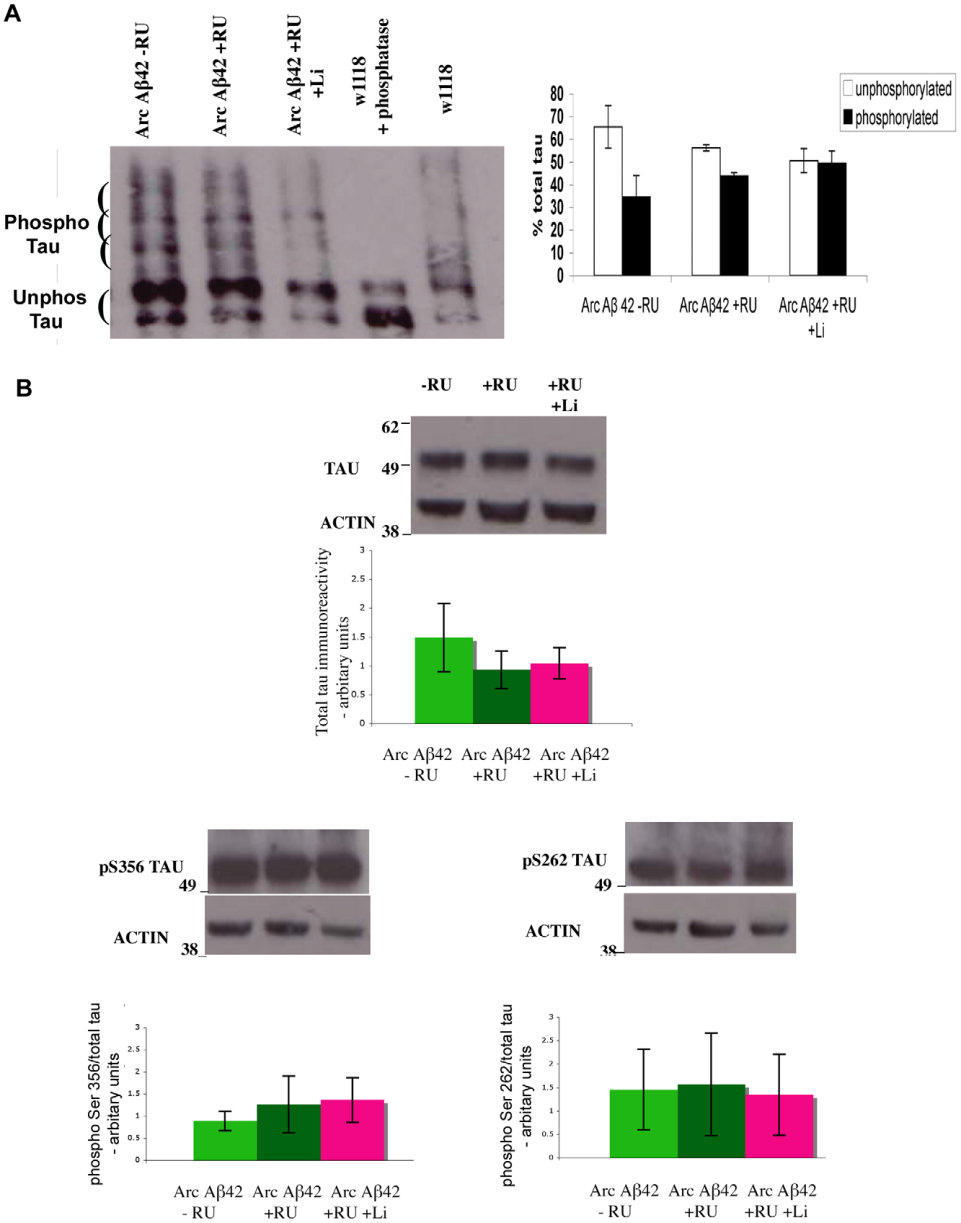
Flies expressing Aβ42 in adult neurons show no changes in total tau phosphorylation and at Ser 262 and Ser 356 specific epitopes. (A) Western blot analyses for total tau phosphorylation using phos-tag gels and (B) phospho Ser262 and Ser356. Tau showed no changes in phosphorylation in flies expressing Aβ42 (UAS-ArcAβ42/GFP;elavGS/+) in comparison to their −RU controls, and when the Aβ42 expressing flies were fed lithium (+RU +Li). Flies were collected 17 days post RU induction, and maintained at 27°C. Quantification of the western blot analysis in (A), phospho-tau or non-phospho-tau normalised to total tau per sample (n = 4), and in (B) n = 4 for total tau and n = 3 for the Ser sites depicted in the bar chart, showed no significant differences between ArcAβ42/+;elavGS/+ +RU flies to −RU controls or to ArcAβ42/+;elavGS/+ +RU +Li flies (one-way ANOVA).

Since GSK-3 is a well-established tau kinase, and tau is abnormally phosphorylated in AD, we next investigated whether the protective effect of Sgg inhibition on Aβ42 toxicity in our fly model is mediated via alterations in tau phosphorylation. Hence, we examined the phosphorylation of *Drosophila* tau in flies over-expressing Arctic Aβ42 peptide in the absence or presence of lithium-treatment (Figure 9).

We analysed tau phosphorylation using Phos-tag acrylamide gels, a technique for separating phosphorylated protein isoforms [39], which has been employed previously to investigate the phosphorylation of fly proteins [40]. Phos-tag is a phosphate-binding compound which, when incorporated into polyacrylamide gels, can result in an exaggerated mobility shift for phosphorylated proteins, dependent on the degree of phosphorylation. When heat-stable fly head homogenates were run on Phos-tag gels, several prominent tau bands were detected, implying that endogenous tau is phosphorylated at multiple sites in WT tissue (Figure 9A). De-phosphorylation using λ-protein phosphatase confirmed that the high molecular weight bands were due to tau phosphorylation, and that non-phosphorylated fly tau runs as a doublet (Figure 9A). This method is thus capable of detecting at least some phosphorylation changes on the endogenous fly tau. Using this method, no difference in the level of tau phosphorylation was observed in flies over-expressing Arctic Aβ42 compared to non-expressing controls or to Arctic Aβ42 flies treated with lithium chloride (Figure 9A and Figure S3A).

The phosphorylation sites on fly tau have not yet been extensively characterized. *Drosophila*-specific phosphorylation-dependent antibodies are hence not available for the examination of specific sites. However, several GSK-3 specific sites [41], [42], and sites reported to be altered by Aβ42 peptide [43]–[45], on human tau appear to be conserved in the *Drosophila* tau sequence (Figure S4). Of these, Ser262, and Ser356 phosphorylation-dependent human tau antibodies were found to detect fly tau protein specifically (Figure 9B and Figure S3B). We therefore used these antibodies to examine the effects on tau phosphorylation at these sites. Arctic Aβ42 over-expression, or treatment of Aβ42-expressing flies with lithium, did not modify phosphorylation at the Ser262 or Ser356 homologous tau epitopes (Figure 9B). This suggests that neither Aβ42 nor GSK-3 are predominant *in vivo* regulators of the phosphorylation of these sites on fly tau.

### Loss of Tau reduces adult-onset Aβ42 toxicity

**Figure 10:**
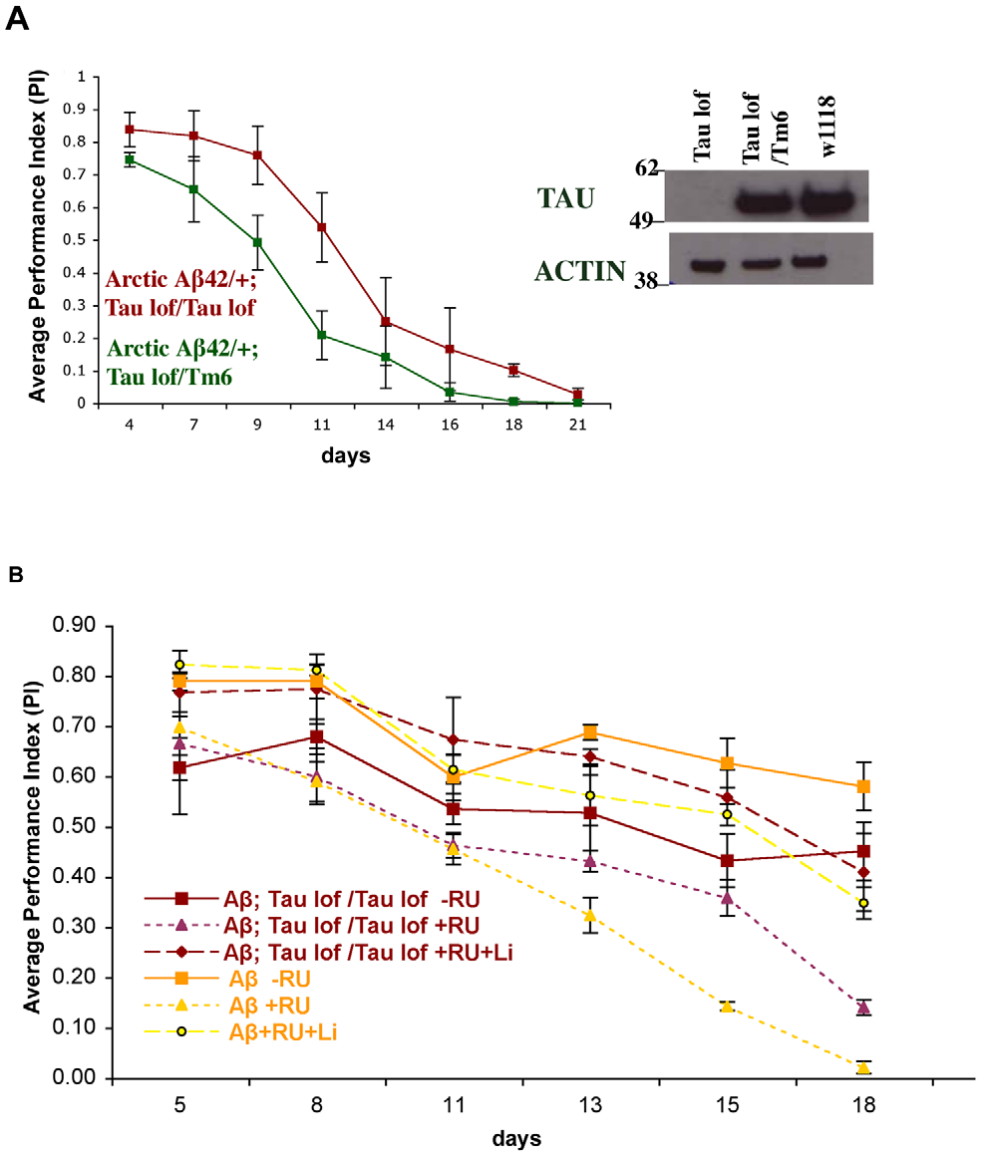
Lithium treatment alone has a greater protective effect against Aβ42 toxicity than loss of tau function alone. (A) Loss of tau partially suppressed the locomotor dysfunction phenotype of Arctic Aβ42 flies. Expression of Arctic Aβ42 peptide in a tau heterozygous background in the adult fly nervous system caused locomotor dysfunction. This phenotype was suppressed when Arctic Aβ42 peptide was expressed in a homozygous tau mutant background. Climbing ability of UAS-ArcAβ42/+;elavGS Tau EP3203/TM6 and UAS-ArcAβ42/+;elavGS Tau EP3203/Tau Dfc flies on +RU486 SY medium was assessed at the indicated time-points (see Materials and Methods). Data are presented as the average performance index (PI) ± SEM (number of independent tests (n) = 3, number of flies per group (n_t_) = 45). *P*<0.0001 comparing the PI of UAS-ArcAβ42/+;elavGS Tau EP3203/Tau Dfc to UAS-ArcAβ42/+;elavGS Tau EP3203/TM6 flies (two-way ANOVA). The crosses were performed at 27°C. Western blotting analysis, using a non-phosphorylation dependent antibody to *Drosophila* tau, confirmed that endogenous tau protein levels were greatly reduced in UAS-ArcAβ42/+;elavGS tau EP3203/tau Dfc flies in comparison to tau heterozygous flies (UAS-ArcAβ42/+;elavGS tau EP3203/TM6) and control w1118 flies. (B) Arctic Aβ42 toxicity was suppressed when the peptide was expressed in a homozygous tau mutant background or when flies were treated with lithium. Lithium treatment alone had a greater protective effect against Aβ42 toxicity than did loss of tau function alone, and was not dependent on tau. Climbing ability of UAS-ArcAβ42/+;elavGS/+ and UAS-ArcAβ42/+;elavGS tau EP3203/tau Dfc flies on − or +RU486 SY medium, in the presence or absence of lithium, was assessed at the indicated time-points. Data are presented as the average PI ± SEM and were analysed by two-way ANOVA (number of independent tests (n) = 4, number of flies per group (n_t_) = 60). Comparing PI of UAS-ArcAβ42/UAS-gfp;elavGS/+ and UAS-ArcAβ42/+;elavGS tau EP3203/tau Dfc flies on −RU, significant differences between genotypes (*P*<0.0001) were observed. *P* = 0.0002 comparing PI of UAS-ArcAβ42/UAS-gfp;elavGS/+ and UAS-ArcAβ42/+;elavGS tau EP3203/tau Dfc flies on +RU food. No significant differences were observed comparing UAS-ArcAβ42/+;elavGS/+ and UAS-ArcAβ42/+;elavGS tau EP3203/tau Dfc flies on +RU +lithium (*P* = 0.692).

Although we could not uncover a role for tau phosphorylation in Aβ42 toxicity, we investigated whether the presence of tau modulates Aβ42 pathology. We found that loss of tau reduced the Arctic Aβ42 climbing dysfunction. UAS-ArcAβ42/+;elavGS tau EP3203/tau deficiency (dfc) flies, which express Arctic Aβ42 in a genetic background homozygous mutant for tau (see Figure 10A for tau expression levels; tau antibody has previously been described [46]), had improved locomotor ability compared to UAS-ArcAβ42/+;elavGS tau EP2303/TM6 flies, which express a much greater level of tau (*P*<0.0001, two-way ANOVA; Figure 10A) and UAS-ArcAβ42/+;elavGS/+ flies, which express wild-type tau levels (*P* = 0.0002, two-way ANOVA; Figure 10B), on +RU food. It is important to note, however, that tau loss of function flies themselves displayed some locomotor dysfunction compared to controls (Figure 10B; *P*<0.0001 comparing UAS-ArcAβ42/+;elavGS/+ to UAS-ArcAβ42/+;elavGS tau EP3203/tau dfc on −RU486, two-way ANOVA), thus potentially reducing the apparent protective effect of removing tau on Aβ42 pathology. These data parallel observations in mammals, where loss of murine Tau rescued Aβ-induced behavioural deficits in a mouse AD model [8].

We further investigated interactions between *Drosophila* tau and GSK-3 in the protection against Aβ42 pathology, to determine if they might act in the same biochemical pathway (Figure 10B). Lithium treatment alone had a greater protective effect against Aβ42 toxicity than did loss of tau function alone, although this may have been confounded by the reduced climbing ability of flies with reduced tau. Moreover, lithium treatment rescued Aβ42-induced climbing dysfunction to the same extent in the presence or absence of tau, (*P* = 0.692 comparing UAS-ArcAβ42/+;elavGS/+ and UAS-ArcAβ42/+;elavGS tau EP3203/tau dfc flies on +RU, + lithium food, two-way ANOVA) suggesting that endogenous tau is not required for the lithium effect. This suggests that a large proportion of the protective effect of GSK-3 inhibition on Aβ42 toxicity is mediated via non-tau-dependent mechanisms. We also show that tau is required for the manifestation of Aβ42 effects. Our experimental design does not address, neither excludes, the possibility that a direct interaction of GSK-3 and tau may also affect Aβ42 toxicity.

### Inhibition of Shaggy in the adult nervous system reduces Aβ load in Arctic Aβ42-expressing flies

**Figure 11:**
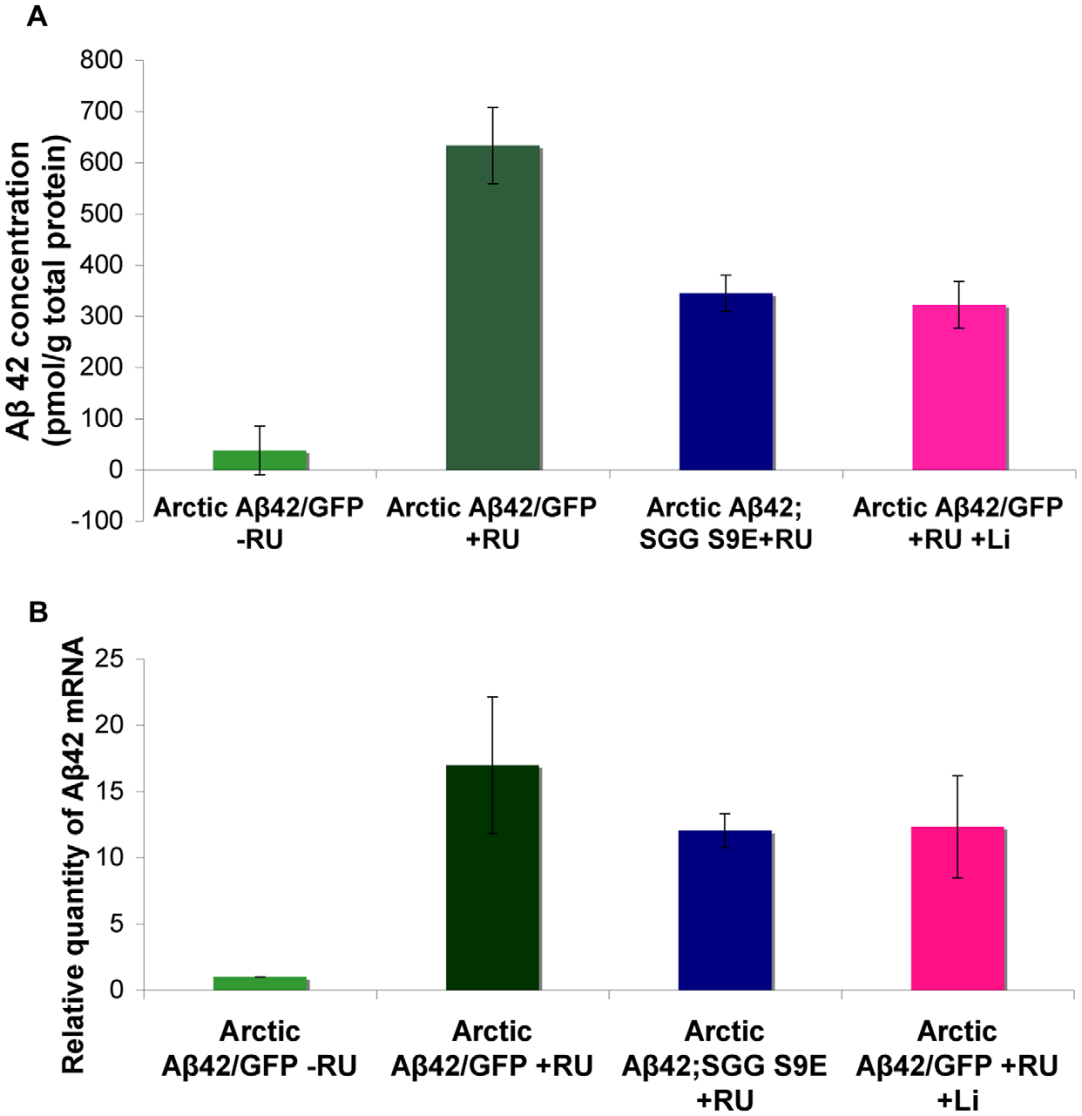
Inhibiting Shaggy activity reduces amyloid levels of Arctic Aβ42 flies. (A) Protein levels of UAS-ArcAβ42/UAS-gfp;elavGS/+ and UAS-ArcAβ42/+;elavGS/UAS-SggS9E flies on +RU486 SY medium, and UAS-ArcAβ42/UAS-gfp;elavGS/+ flies on +RU +Lithium (Li), were measured by ELISA at 15 days post-induction (see Materials and Methods). Data were compared using one-way ANOVA, number of independent tests (n) = 3. P<0.01, and P<0.0001 when comparing UAS-ArcAβ42/UAS-gfp;elavGS/+ to UAS-ArcAβ42/+;elavGS/UAS-SggS9E flies on +RU486 SY medium, and UAS-ArcAβ42/UAS-gfp;elavGS/+ +RU to UAS-ArcAβ42/UAS-gfp;elavGS/+ +RU +Li respectively. (B) RNA levels of UAS-ArcAβ42/UAS-gfp;elavGS/+, UAS-ArcAβ42/+;elavGS/UAS-SggS9E +RU486 SY medium and UAS-ArcAβ42/UAS-gfp;elavGS/+ +RU + lithium flies were measured at day 5. No significant difference was seen in the levels of RNA expression, number of independent tests (n) = 3.

Because the amelioration of Aβ42 toxicity by reduced GSK-3 activity did not appear to be mediated mainly through tau, we next examined the direct effect of GSK-3 inhibition on Aβ42 levels. Interestingly, we found by ELISA analysis that Aβ peptide was significantly reduced in flies expressing Arctic Aβ42 when Sgg activity was reduced. Adult flies that co-expressed Arctic Aβ42 and the inactive dominant negative mutant Sgg S9E, or adult flies that expressed Arctic Aβ42 and were fed lithium, showed a major reduction in total Aβ42 levels in comparison to flies expressing Arctic Aβ42 alone and reared on food without lithium (Figure 11A). The transcript levels of Aβ42 in the presence of functional or inhibited Sgg activity were not significantly different (Figure 11B), suggesting that Sgg does not affect transgene expression, but rather acts directly or indirectly on Aβ degradation/sequestration. These data demonstrate for the first time a role of GSK-3 in determining the level of Aβ42 peptide, in the absence of effects on APP processing. Furthermore, this reduction in the levels of Aβ42 peptide by GSK-3 inhibition is most likely not mediated by tau, since loss of tau function partially rescued Aβ toxicity, but did not affect Aβ42 levels (Figure 10A and Figure S5). These data again suggest that GSK-3 can modify Aβ42 toxicity via tau-independent mechanisms.

## Discussion

Glycogen synthase kinase-3 is increasingly thought to play a pivotal role in the pathogenesis of Alzheimer's disease, both as a regulator of the accumulation of Aβ peptide [12], [17], [47] and through its well-established role as a tau kinase [48]–[52]. Although previous studies in mice have suggested that GSK-3 alters Aβ levels via modulation of APP processing [12], [17], the direct effects of the enzyme on Aβ toxicity, and in the adult nervous system, have not been examined. We therefore performed a more direct analysis of the specific role of GSK-3 in regulating Aβ42 toxicity in adult neurons *in vivo*, by modulating its activity in an adult-onset *Drosophila* model of Alzheimer's disease. Our study shows for the first time that GSK-3 inhibition ameliorates Aβ42 toxicity in adult flies, and also highlights a novel mechanism of protection by which GSK-3 directly regulates Aβ42 levels in the absence of any effects on APP processing.

We have generated an inducible *Drosophila* model of Alzheimer's disease. Over-expression of the Arctic Aβ42 peptide in adult fly neurons led to shortened lifespan, neuronal dysfunction and behavioural impairments. However, no neuronal loss was evident in the brains of these flies. This finding contrasts with previous reports that flies over-expressing Arctic Aβ42, using a constitutive neuronal driver, develop vacuoles [28], [33], These contrasting results could be reconciled if neuronal loss upon Aβ42 over-expression is a consequence of developmental abnormalities in the *Drosophila* brain or if neuronal loss represents an end-stage event in response to Aβ42 toxicity, since vacuolation has been reported only under the most extreme conditions of expression, age and temperature, while neuronal toxicity in these models is already apparent under less stringent conditions [33]. Importantly, our findings agree with those of other studies demonstrating that neuronal loss is generally not evident in murine models of amyloidosis, such as in mice transgenic for the amyloid precursor protein [53]. Moreover, our study has provided direct evidence of neuronal dysfunction in response to Aβ42, by electrophysiological methods, at a timepoint more suitable to understanding the early events that lead to neuronal decline in AD.

GSK-3 activity was increased in our flies upon Arctic Aβ42 over-expression, as measured by reductions in phosphorylation of endogenous Sgg at the inhibitiory Ser9 site. This is consistent with previous observations showing that Aβ42 alters GSK-3 phosphorylation in cells [54] and in mice [14]. Contrary to these findings, however, one other study has shown that WT Aβ42 expression does not alter phosphorylation of Sgg in flies [55]. These differences may reflect varying mechanisms by which Arctic mutant Aβ42 and WT Aβ42 peptides modulate GSK-3 activity, or a distinction in the effect of expressing Aβ42 throughout development compared to adult-only expression. Hence, our observed increase in Sgg activity may reflect an age-dependent effect. We aimed, therefore, to further investigate the functional role of this kinase in mediating Aβ toxicity in our adult-onset AD model.

GSK-3 plays an important role in neuronal development [56], [57]. Previous analyses of the role of GSK-3 in amyloid toxicity using constitutive expression systems may, therefore, represent abnormal neuronal development in addition to the response to AD pathology in the adult period. Hence, we confined GSK-3 inhibition to the adult neurons of Arctic Aβ42-expressing flies, by over-expressing a dominant negative form of the *Drosophila* orthologue Sgg, using our inducible expression system, or we treated whole adult flies with the GSK-3 inhibitor, lithium. GSK-3 inhibition extended lifespan of Arctic Aβ42-expressing flies and suppressed the locomotor dysfunction caused by expression of the peptide in adult neurons. This is an important finding because it demonstrates a definitive role for GSK-3 in Aβ42 pathogenesis in adult flies. Moreover, we found that inhibiting Sgg in adulthood had no adverse effect on wild type flies. Hence, our study provides further support for the therapeutic potential of GSK-3 inhibitors in treating Alzheimer's disease. Further studies are required to test this potential in mammalian models, firstly to confirm the relevance of GSK-3 for direct regulation of Aβ42 toxicity and secondly to establish a therapeutic index for GSK-3 inhibition in the treatment of AD.

Aβ42 has been shown previously to increase tau phosphorylation in cells [45] and in mice [5], [43]. Correlative evidence has suggested that GSK-3 may mediate the effects of Aβ on tau phosphorylation, since Aβ increases GSK-3 activity [10]. Furthermore, GSK-3 and APP cause similar increases in tau phosphorylation and aggregation in tau over-expressing mice [14]. Because both tau and GSK-3 appeared to play a causal role in Arctic Aβ42 toxicity in our flies, we examined whether the protective effect of GSK-3 inhibition on Aβ toxicity might be mediated via alterations in phosphorylation of endogenous *Drosophila* tau. We found that Arctic Aβ42 over-expression and lithium treatment of Aβ42-expressing flies did not have an observable effect on overall tau phosphorylation, as revealed both by generic measures, and at two specific sites by examination of phosphorylation of fly tau at Ser262 and Ser356 epitopes. However, because the Ser 262 and 356 sites are also predominant *in vivo* substrates for MARK (microtubule-associated protein (MAP)-microtubule affinity regulating kinase) [58], [59], further investigation of more specific GSK-3 sites on *Drosophila* tau, using mass spectrometric methods, would provide a more definitive analysis of the role of tau phosphorylation by GSK-3 in mediating Aβ42 toxicity in the fly.

Our data suggest that tau phosphorylation may not be the only mechanism by which Aβ42 exerts its toxic effect in *Drosophila*, since phosphorylation sites thought to be important for mediating Aβ42 effects on tau (Thr212/Ser214 [43], Thr231 [45], Ser422, Ser262 [44]) are predominantly not conserved in the fly, or are not altered by Aβ42 over-expression in our AD model. These findings seem to contradict previous studies showing that Aβ42 increases phosphorylation of human tau in *Drosophila* [55], [60], and indicating that particular sites, such as Ser262, are important in mediating Aβ toxicity in the fly [60]. This disparity could reflect a lack of conservation of the mechanisms through which Aβ42 regulates human and *Drosophila* tau proteins. However, the endogenous fly tau does appear to be an important downstream mediator of Aβ42 toxicity, since loss of tau function partially reduced Aβ42 pathology in our study. This protective effect of tau against Aβ42 toxicity, however, may have been masked by the locomotor dysfunction from reducing levels of tau itself in flies that do not over-express the Aβ42 peptide. As a previous study has reported similar tau-dependent neuropathological phenotypes in an APP-overexpressing mouse model of AD [8], our findings suggest that the role of tau in amyloid toxicity is conserved over large evolutionary distances.

We further examined the epistatic interaction between GSK-3 inhibition and tau loss of function in protecting against Aβ42 toxicity in our fly model. Lithium alone had a greater protective effect against Aβ42 toxicity than loss of tau function alone, and lithium could prevent Aβ42-induced dysfunction in the presence or absence of tau. This suggests that a large proportion of the protective effect of GSK-3 inhibition on Aβ42 toxicity is mediated via non-tau-dependent mechanisms and that tau is not required for this effect. We observed no additive effect of combining lithium treatment and tau loss of function in protection against Aβ42-induced pathology; however, this could have been a consequence either of toxicity of loss of tau in the absence of Aβ42 or of the level of protection afforded by lithium, which could have produced a ceiling effect. Our findings agree with other recently published studies, showing that loss of tau only partially protects against GSK-3-induced neuronal degeneration in adult mice [61], suggesting that other non-tau-dependent mechanisms of GSK-3 neuro-toxicity exist.

Previous studies have shown a reduction in Aβ load in mice as a result of GSK-3 inhibition, but this has been explained mainly by dysregulation of APP processing, either by increasing γ-secretase activity [12] or by increasing the phosphorylation of APP and therefore directing its subcellular location to sites of secretase activity [17]. Our inducible *Drosophila* model, however, expresses the Arctic Aβ42 peptide directly, thus circumventing the requirement for APP processing. We found that inhibition of GSK-3 caused a reduction in the level of Aβ42 peptide, but not in RNA transcript levels. Hence, although previous studies have indicated that GSK-3 does not affect Aβ degradation [17], our findings demonstrate a novel effect of GSK-3 in Aβ metabolism, irrespective of APP processing, in the adult nervous system. Furthermore, this observation may, partially, explain the non-tau-dependent effect of GSK-3 in protecting against Aβ toxicity in our flies.

Our study, therefore, implies that GSK-3 may increase Aβ degradation or clearance. Candidate *in vivo* Aβ degrading enzymes include neprilysin (NEP) [62], insulin degrading enzyme (IDE) [63]–[64] and to a lesser extent endothelin converting enzymes (ECE-1,2) [65] and plasmin [66]. Mice deficient in IDE [63], [64] or NEP [62] display increased levels of Aβ peptides in the brain. Conversely, increasing expression and activity of NEP or IDE reduces the cerebral amyloid plaque burden observed in APP over-expressing mice [67], further implying that these are predominant *in vivo* Aβ degrading enzymes. Direct interactions between GSK-3 and IDE or neprilysin activities in relation to Aβ degradation, however, have not been extensively investigated. Studies examining the effects of reduced insulin signalling on amyloid toxicity in APP over-expressing mice have reported either increased GSK-3 activity, reduced IDE activity and increased amyloidosis [68] or decreased GSK-3 activity, increased IDE expression and reduced amyloidosis [69] in the brain, suggesting an inverse correlation between GSK-3 and IDE activities in relation to Aβ metabolism. Other studies have reported no correlation between inhibition of GSK-3 activity and neprilysin levels in relation to reduced Aβ load in mice [17], but NEP activity was not measured and may provide a more accurate indication of the role of this enzyme in Aβ metabolism downstream of GSK-3. As most information from mouse models is correlative, however, further work is required to determine whether these degrading enzymes are direct mediators of Aβ degradation in response to GSK-3 inhibition, by modulating their activities in our inducible *Drosophila* model. *Drosophila* homologues of both IDE and NEP exist, and over-expression of both NEP [26], [70] and IDE [71] have been shown to reduce Aβ induced neurotoxicity in flies. This suggests that these degradation mechanisms may be generally conserved, and that the fly is a valuable model for the direct analysis of these genetic interactions with respect to the role of GSK-3 in AD.

The more general proteosome degradation pathway could also play a role in regulating Aβ degradation or sequestration. Heat shock protein 90 (Hsp90), a protein chaperone involved in the proteosome degradation pathway, is thought to phosphorylate GSK-3 and regulate its activity [13]. In addition, an increase in levels of GSK-3 down-regulates the transcriptional activity of Heat shock factor-1 (HSF-1) and Hsp70 [72]; thus a decrease in GSK-3 activity could lead to an increase in levels/activity of these chaperone molecules and augment the levels of Aβ. In a *Caenorhabdits elegans* worm model of AD, the aggregation-mediated Aβ42 toxicity was regulated by modulating the levels of hsf-1; a reduction in hsf-1 increased paralysis in these worms, suggesting a role of hsf-1 in the dis-aggregation of Aβ toxic oligomers [73]. Thus, any of these pathways/molecules could play a role in affecting Aβ load in our fly model and will require detailed exploration in the future.

Our data highlight that this fly model is suitable for the study of AD pathology, since we observe neuronal dysfunction and toxicity that are particular to the expression of Aβ42 peptide. Furthermore, we have been able to test the amyloid cascade hypothesis in part, to show that tau is acting downstream of Aβ pathology. This inducible model of AD will also open the way to understanding the role of events at different ages and of the ageing process itself in the biological pathway leading to this ageing related disease. We have shown the involvement of GSK-3, particularly in adulthood, in AD pathogenesis, and also uncover a novel mechanism by which GSK-3 could be acting directly or indirectly on Aβ, by reducing Aβ load. These results raise new potential therapeutic benefits of GSK-3 in AD pathology.

## Materials and Methods

### Fly stocks and maintenance

All fly stocks were maintained at 25°C or 27°C on a 12∶12-h light∶dark cycle at constant humidity on a standard sugar-yeast (SY) medium (15gl^−1^ agar, 50 gl^−1^ sugar, 100 gl^−1^ autolysed yeast, 100gl^−1^ nipagin and 3ml l^−1^ propionic acid). Adult-onset neuronal-specific expression of Arctic mutant Aβ42 peptide or constitutively active Sgg was achieved by using the elav GeneSwitch (elavGS)-UAS system [GAL4-dependant upstream activator sequence; [30]]. ElavGS was derived from the original elavGS 301.2 line [30] and obtained as a generous gift from Dr H. Tricoire (CNRS, France). UAS-ArcAβ42 and UAS-SggS9E were obtained from Dr D. Crowther (University of Cambridge, UK) and the Bloomington Drosophila Stock Centre respectively. Tau dfc (9530) and EP line 3203 (17098) were received from Bloomington Drosophila stock centre. The EP line orientation is opposite to tau expression and causes a reduction in tau expression [46]. elavGS and UAS-lines used in all experiments were backcrossed six times into the w1118 genetic background. Male flies expressing UAS-constructs were crossed to female flies expressing elavGS, and adult-onset neuronal expression induced in female progeny by treatment with mifepristone (RU486;200mM) added to the standard SY medium.

### Lithium treatment protocol

Lithium Chloride was made at 1M concentration and added to 200mM RU486 standard SY medium at a final concentration of 30mM or 100mM.

### Lifespan analyses

For all experiments, flies were raised at a standard density on standard SY medium in 200 mL bottles. Two days after eclosion once-mated females were transferred to experimental vials containing SY medium with or without RU486 (200mM) at a density of 10 flies per vial. Deaths were scored almost every other day and flies were transferred to fresh food three times a week. Statistical analyses were performed using JMP (version 7.0) software (SAS Institute, Cary, NC, USA). Data are presented as survival curves and analysis was performed using log-rank tests to compare between groups.

### Negative Geotaxis Assays

To characterise the adult-onset behavioural effects of Arctic Aβ42 peptide on neuronal function, climbing assays were initially performed at 25°C according to previously published methods [75]. Climbing ability was analysed every 2–3 days post-RU486 treatment. Briefly, 15 adult flies were placed in a vertical column (25cm long, 1.5cm diameter) with a conic bottom end, tapped to the bottom of the column, and their subsequent climb to the top of the column was analysed. Flies reaching the top and flies remaining at the bottom of the column after a 45 sec period were counted separately, and three trials were performed at 1 min intervals for each experiment. Scores recorded were the mean number of flies at the top (n_top_), the mean number of flies at the bottom (n_bottom_) and the total number of flies assessed (n_tot_). A performance index (PI) defined as ½(n_tot_+n_top_−n_bottom)_/n_tot_) was calculated. Data are presented as the mean PI ± SEM obtained in three independent experiments for each group, and analysis of variances (ANOVA) and post hoc analyses were performed using JMP 7.0 software.

To assess various modifiers of the adult-onset neuronal dysfunction in Arctic Aβ42 over-expressing flies, a less stringent climbing assay was performed at 27°C. Thirty flies expressing Arctic Aβ42 with or without co-expressing modifiers in the neurons (elav-GAL4GS) were used for the climbing assay, adapted from Ganetzky *et al.* [76]. Climbing performance (ability to climb past a 5cm mark in 18s) was assessed after eclosion post RU486 treatment.

### Electrophysiology

Recordings from the GFS of adult flies were made essentially as described in [77]; a method based on those described by Tanouye and Wyman (1980) [78] and Gorczyca and Hall (1984) [79]. Flies were anaesthetized by cooling on ice and secured in wax, ventral side down, with the wings held outwards in the wax. A tungsten earth wire (ground electrode) was placed into abdomen; tungsten electrodes were pushed through the eyes and into the brain to deliver a 40V pulse for 0.03ms using a Grass S48 stimulator. Recordings were made from the TTM and contralateral DLM muscle using glass microelectrodes (resistance: 40–60 MΩ). The electrodes were filled with 3M KCl and placed into the muscles through the cuticle. Responses were amplified using Getting 5A amplifiers (Getting Instruments, USA) and the data digitized using an analogue-digital Digidata 1320 and Axoscope 9.0 software (Axon Instruments, USA). For response latency recordings, at least 5 single stimuli were given with a 5s rest period between each stimulus; trains of 10 stimuli, at either 100Hz, 200 Hz or 250Hz, were given a 5s rest interval between each train.

### Histology

For neuronal cell loss, adult heads were fixed, dehydrated and transverse sections were stained with Toluidine blue. Cell bodies were then counted blind for each genotype.

### Quantitative RT–PCR

Total RNA was extracted from 20–25 fly heads using Trizol (GIBCO) according to the manufacturers' instructions. The concentration of total RNA purified for each sample was measured using an *eppendorf biophotometer*. 1µg of total RNA was then subjected to DNA digestion using DNAse I (Ambion), immediately followed by reverse transcription using the Superscript II system (Invitrogen) with oligo(dT) primers. Quantitative PCR was performed using the PRISM 7000 sequence-detection system (Applied Biosystems), SYBR Green (Molecular Probes), ROX Reference Dye (Invitrogen), and Hot Star Taq (Qiagen, Valencia, CA) by following manufacturers' instructions. Each sample was analysed in triplicate with both target gene (Arctic Aβ42) and control gene (RP49) primers in parallel. The primers for the Aβ transgenes were directed to the 5′ end and 3′ end of the Aβ coding sequence: forward GATCCTTCTCCTGCTAACC; reverse CACCATCAAGCCAATAATCG. The RP49 primers were as follows: forward ATGACCATCCGCCCAGCATCAGG; reverse ATCTCGCCGCAGTAAACG.

### Quantification of total, soluble, and aggregated Aβ42

To extract total Aβ42, five fly heads were homogenised in 50 µl GnHCl extraction buffer (5 M Guanidinium HCl, 50 mM Hepes pH 7.3, protease inhibitor cocktail (Sigma, P8340) and 5mM EDTA), centrifuged at 21,000 g for 5 min at 4°C, and cleared supernatant retained as the total fly Aβ42 sample. Alternatively, soluble and insoluble pools of Aβ42 were extracted using a protocol adapted from previously published methods [80]: 200 fly heads were homogenised in 200 µl tissue homogenisation buffer (250mM sucrose, 20mM Tris base, 1mM EDTA, 1mM EGTA, protease inhibitor cocktail (Sigma) then mixed further with 200µl DEA buffer (0.4% DEA, 100mM NaCl and protease inhibitor cocktail). Samples were centrifuged at 135,000 g for 1 hour at 4°C (Beckman Optima Max centrifuge, TLA120.1 rotor), and supernatant retained as the cytosolic, soluble Aβ42 fraction. Pellets were further resuspended in 400µl ice-cold formic acid (70%), and sonicated for 2×30 sec on ice. Samples were re-centrifuged at 135,000 g for 1 hour at 4°C, then 210 µl of supernatant diluted with 4ml FA neutralisation buffer (1M Tris base, 0.5M Na_2_HPO_4_, 0.05% NaN_3_) and retained as the insoluble, formic acid-extractable Aβ42 fraction.

Total, soluble or insoluble Aβ42 content was measured in Arctic mutant Aβ42 flies and controls using the hAmyloid β42 ELISA kit (HS) (The Genetics Company, Switzerland). Total Aβ42 samples were diluted 1∶100, and soluble versus insoluble Aβ42 samples diluted 1∶10 in sample/standard dilution buffer, then ELISA performed according to the manufacturers' instructions. Protein extracts were quantified using the Bradford protein assay (Bio-Rad protein assay reagent; Bio-Rad laboratories Ltd (UK)) and the amount of Aβ42 in each sample expressed as a ratio of the total protein content (pmoles/g total protein). Data are expressed as the mean ± SEM obtained in three independent experiments for each genotype. ANOVAs and Tukey's-HSD post-hoc analyses were performed using JMP 7.0 software.

### Analysis of tau phosphorylation by Phos-tag SDS-PAGE

Fly heads were homogenised in Mes buffer (100 mM Mes, pH 6.5, 1 M NaCl, 0.5 mM MgCl_2_, 1 mM EGTA, 10 mM NaF, Protease inhibitor cocktail [Sigma, P8340] and Phosphatase inhibitor cocktail 2 [Sigma, P5726]), centrifuged at 20,000 g for 30 minutes at 4°C, then supernatants adjusted to 0.5% β-mercaptoethanol, boiled for 5 minutes and re-centrifuged. Cleared supernatants were then retained as heat-stable soluble tau. Control samples were dephosphorylated by incubating with λ-protein phosphatase (NEB, P0753) for 3 hours at 30°C.

To detect phosphorylated and non-phosphorylated tau, samples were separated by SDS-PAGE, using Phos-tag AAL-107 (FMS laboratory) according to the manufacturers' instructions. Western blotting was then performed using a non-phosphorylation dependent *Drosophila* rabbit anti-tau antibody (1∶5000). Quantitation was performed using Image J software (National Institutes of Health). Phosphorylated and non-phosphorylated tau was expressed as a percentage of the total amount of tau present in each sample. ANOVA and Tukey's-HSD post-hoc analyses were performed using JMP 7.0 software.

### Western blotting

*Drosophila* heads were pooled and homogenised in 2×Laemmli sample buffer containing β-mercaptoethanol and boiled for 10 mins. Proteins were separated on SDS polyacrylamide gels and blotted onto nitrocellulose. Membranes were incubated in a blocking solution containing 5% milk proteins either in TBST for 1hr at room temperature for Tau blot or in PBST for 20 min for Sgg blot, then probed with primary antibodies overnight at 4°C. Mouse anti-actin antibody (Santacruz) was used at a 1 in 1000 dilution. Tau antibody (rabbit) was a kind gift from Nick Lowe, and used at a 1 in 2000 dilution. Rabbit anti-phospho S262 and S356 (Abcam) were used at a dilution of 1∶1000 in 5% BSA TBST. Quantitation was performed using Image J software. Rabbit anti-phospho S9/S21 GSK-3 antibody (AB 9331 Cell Signaling) at a 1 in 250 dilution was detected using an anti-rabbit 800 nm flourophore conjugate (Rockland, USA). Monoclonal antibody, pan Sgg mouse monoclonal (4G1G) at a 1 in 2000 dilution was a kind gift from Marc Bourouis, which was subsequently detected using a 680 nm anti-mouse flourophore conjugate (Invitrogen, UK). Membranes were sequentially scanned at 700 and 800 nm using a Licor Odyssey infrared scanner. Densitometric measurements were taken in both wavelengths. Relative phospho-serine 9 Sgg levels were determined by dividing the signal at 800 nm by that obtained at 700 nm. Details of secondary antibodies and Odyssey analysis have been previously described in [69].

## Supporting Information

Figure S1 Neuronal electrophysiology of flies over-expressing Arctic Aβ42 peptide in adulthood. Representative traces for (A) TTM and DLM response latencies and (B) TTM responses to high frequency stimulation (100 Hz) measured in elavGS/+;UAS-ArcAβ42/+ flies fed with + or − RU486 medium at days 16 and 28. TTM and DLM response latency was increased in Arctic Aβ42 over-expressing flies at day 28, but not at day 16 (marked with arrows). Vertical scale bars, 50 mV (TTM) and 60mV (DLM) for response latencies, 20 mV for following at 100Hz; horizontal, 2 ms for response latencies, 20ms for following at 100 Hz.
(0.11 MB DOC)

Figure S2 Neuronal cell loss is not evident in flies over-expressing Arctic Aβ42 peptide in adulthood. Cell loss was quantified at day 21 in elavGS/+;UAS-ArcAβ42/+ flies, fed with + or − RU486 medium and maintained at 27 degrees, by counting the number of cell bodies per brain hemisphere. No significant difference was observed when the two genotypes were compared (student's t-test), N = 7 per genotype.
(0.22 MB DOC)

Figure S3 Investigating tau phosphorylation. (A) Western blot analyses of tau phosphorylation using phos-tag polyacrylamide gels. Tau showed no changes in phosphorylation in flies expressing Aβ42 (UAS-ArcAβ42/GFP;elavGS/+) in comparison to their −RU controls, and when the Aβ42 expressing flies were fed lithium (+RU +Li). (B) Phospho-Ser262 and Ser356 human tau antibodies specifically detect tau in fly head homogenates. The 55 kDa tau band is reduced in tau lof/TM6 and absent in tau lof/tau lof flies compared to w1118 controls.
(1.23 MB DOC)

Figure S4 Full-length Tau (P10636), the longest CNS Tau isoform (P10636-8) and *Drosophila* Tau were aligned by Clustal W alignment. *Shaded in sky and light blue are sites conserved between human and Drosophila tau that are phosphorylated by GSK-3 in vitro. The two Ser sites 262 and 356 (light blue) had available human tau antibodies that detect those conserved sites in *Drosophila*. ** Shaded in red are likely GSK-3 sites and Abeta sites (212 and 214 for Abeta) that we had available antibodies for and tested in *Drosophila*, but did not work since they are not conserved. ***Shaded in yellow is an Abeta site (Ser 422) that is conserved in *Drosophila*, which we had an antibody for; Ser 262 is also a likely Abeta site.
(0.03 MB DOC)

Figure S5 Reduction in tau levels does not affect amyloid levels of Arctic Aβ42 flies. Protein levels of UAS-ArcAβ42/+;elavGS Tau EP3203/Tau Dfc to UAS-ArcAβ42/+;elavGS Tau EP3203/TM6 flies flies on +RU486 SY medium, were measured by ELISA at 21 days post-induction. Data are presented as the average protein concentration, ± standard error of mean, data were compared using one-way ANOVA and student t-test, number of independent tests (n) = 3. No significant difference was seen in the levels of Aβ42.
(0.08 MB DOC)
