# Correction: Inhibition of GSK-3 Ameliorates Aβ Pathology in an Adult-Onset *Drosophila* Model of Alzheimer's Disease

**DOI:** 10.1371/annotation/baa8a2a9-130b-4959-b6fb-6f786fd02826

**Published:** 2012-01-05

**Authors:** Oyinkan Sofola, Fiona Kerr, Iain Rogers, Richard Killick, Hrvoje Augustin, Carina Gandy, Marcus J. Allen, John Hardy, Simon Lovestone, Linda Partridge

In Figure 4, the results labelled TTM should be labelled DLM and vice versa. Please view the correct figure here: 

**Figure:**
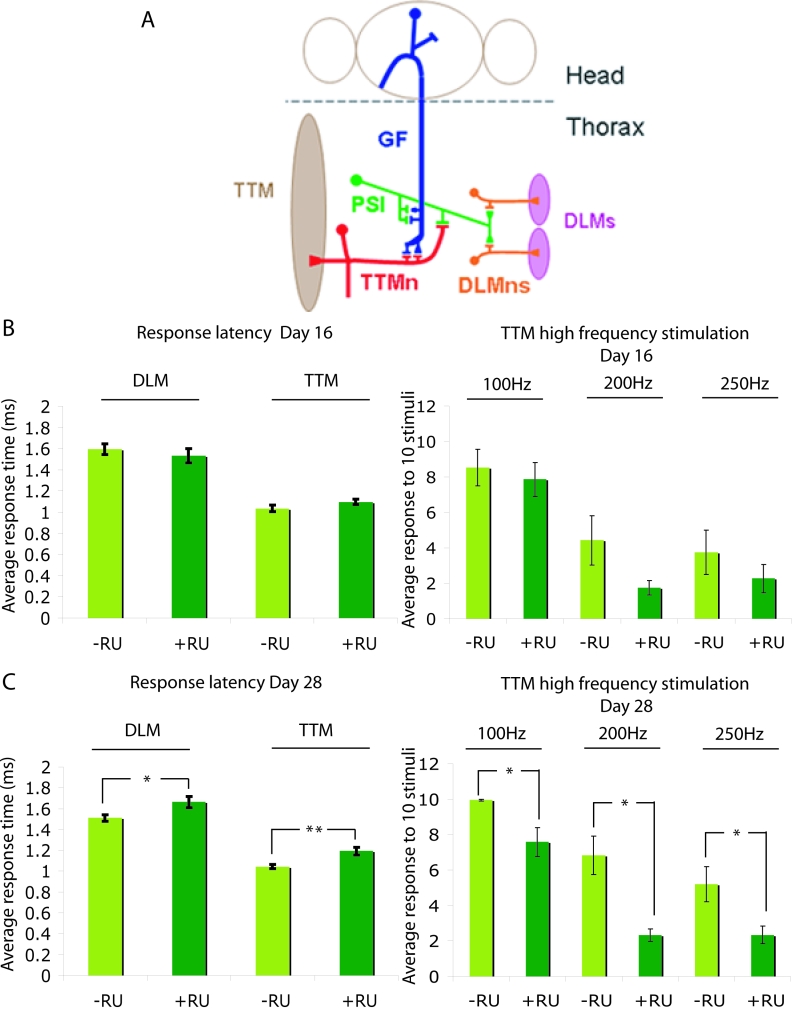


. 

