# Supplementary figures and images for: Inhibition of GSK-3 Ameliorates Aβ Pathology in an Adult-Onset Drosophila Model of Alzheimer's Disease
Source: PLoS Genet. 2010 Sep 2;6(9):e1001087. doi: 10.1371/journal.pgen.1001087 (PMC2932684; doi:10.1371/journal.pgen.1001087)

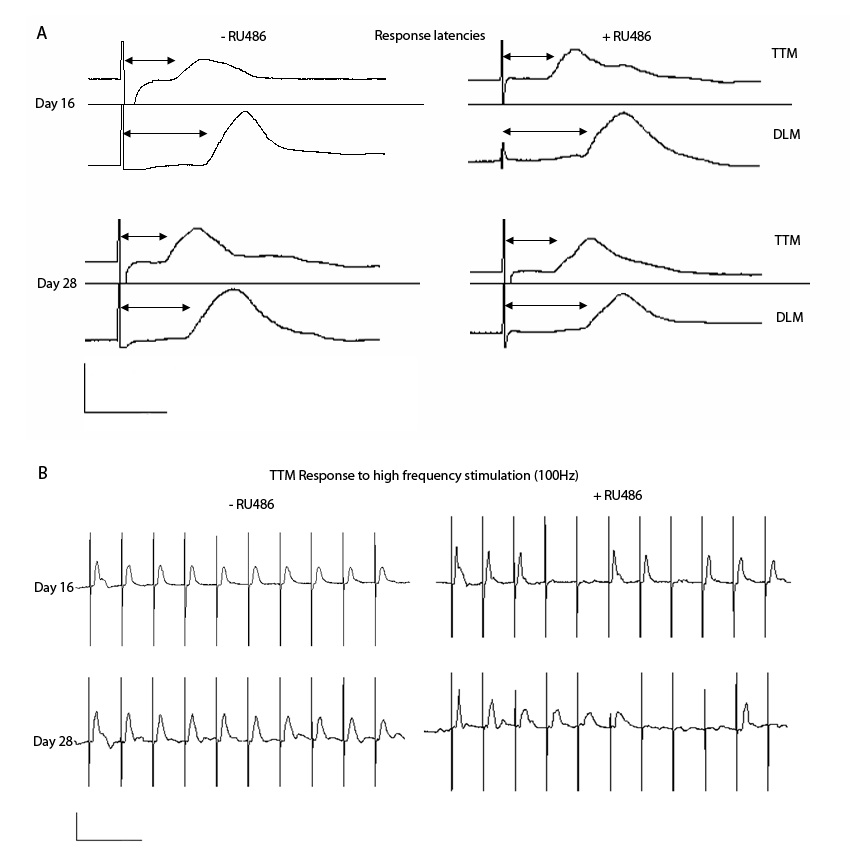


Supplementary Figure 1

Supplement: Figure S1 — Neuronal electrophysiology of flies over-expressing Arctic Aβ42 peptide in adulthood. Representative traces for (A) TTM and DLM response latencies and (B) TTM responses to high frequency stimulation (100 Hz) measured in elavGS/+;UAS-ArcAβ42/+ flies fed with + or − RU486 medium at days 16 and 28. TTM and DLM response latency was increased in Arctic Aβ42 over-expressing flies at day 28, but not at day 16 (marked with arrows). Vertical scale bars, 50 mV (TTM) and 60mV (DLM) for response latencies, 20 mV for following at 100Hz; horizontal, 2 ms for response latencies, 20ms for following at 100 Hz. (0.11 MB DOC) [file pgen.1001087.s001.doc]

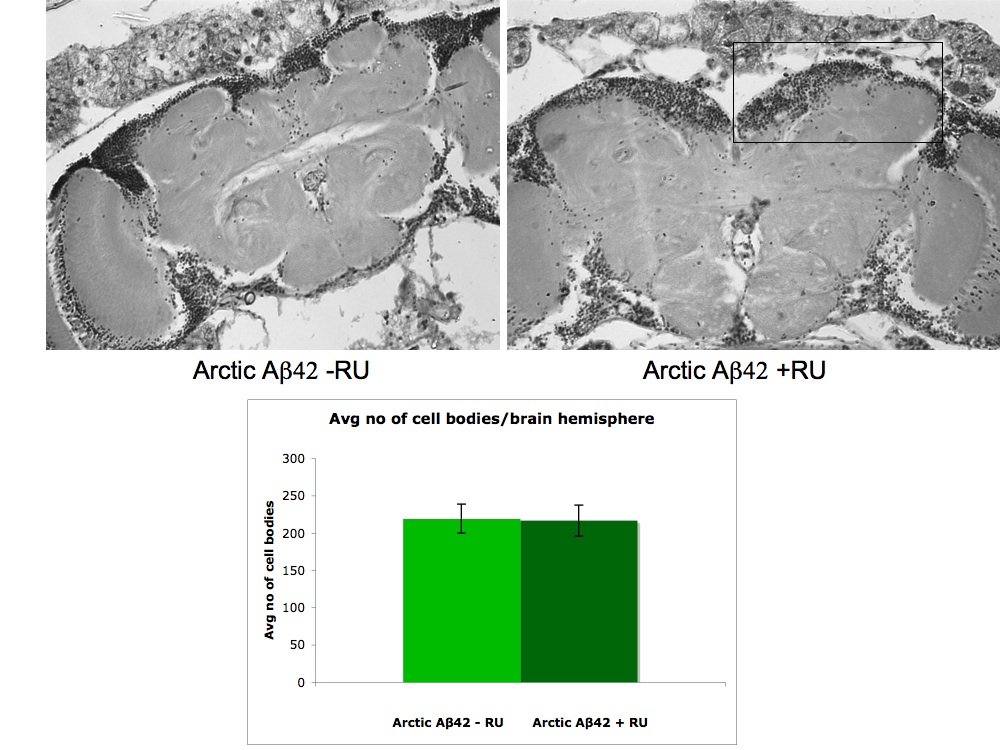


Supplementary Figure 2

Supplement: Figure S2 — Neuronal cell loss is not evident in flies over-expressing Arctic Aβ42 peptide in adulthood. Cell loss was quantified at day 21 in elavGS/+;UAS-ArcAβ42/+ flies, fed with + or − RU486 medium and maintained at 27 degrees, by counting the number of cell bodies per brain hemisphere. No significant difference was observed when the two genotypes were compared (student's t-test), N = 7 per genotype. (0.22 MB DOC) [file pgen.1001087.s002.doc]

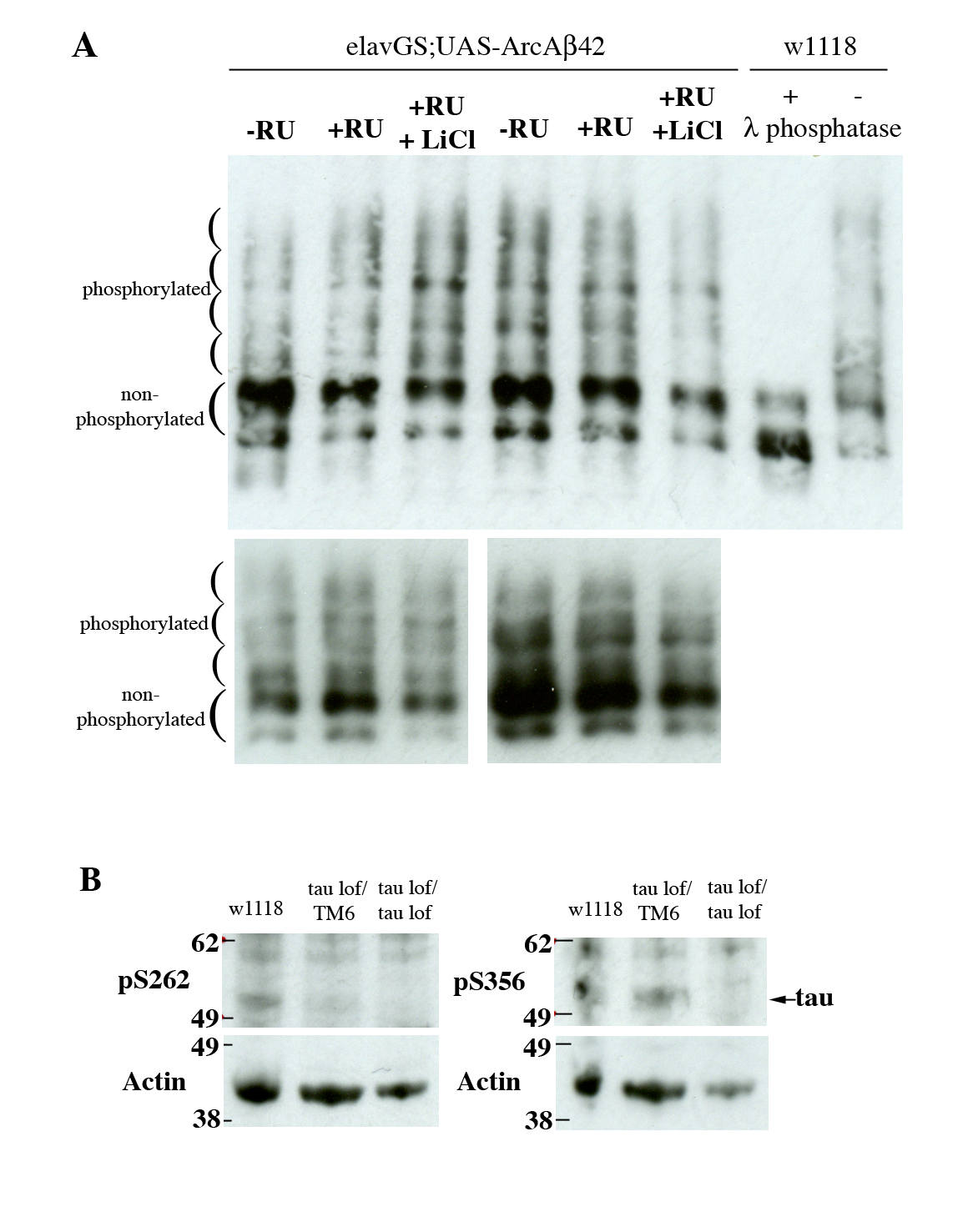


Supplementary Figure 3

Supplement: Figure S3 — Investigating tau phosphorylation. (A) Western blot analyses of tau phosphorylation using phos-tag polyacrylamide gels. Tau showed no changes in phosphorylation in flies expressing Aβ42 (UAS-ArcAβ42/GFP;elavGS/+) in comparison to their −RU controls, and when the Aβ42 expressing flies were fed lithium (+RU +Li). (B) Phospho-Ser262 and Ser356 human tau antibodies specifically detect tau in fly head homogenates. The 55 kDa tau band is reduced in tau lof/TM6 and absent in tau lof/tau lof flies compared to w1118 controls. (1.23 MB DOC) [file pgen.1001087.s003.doc]

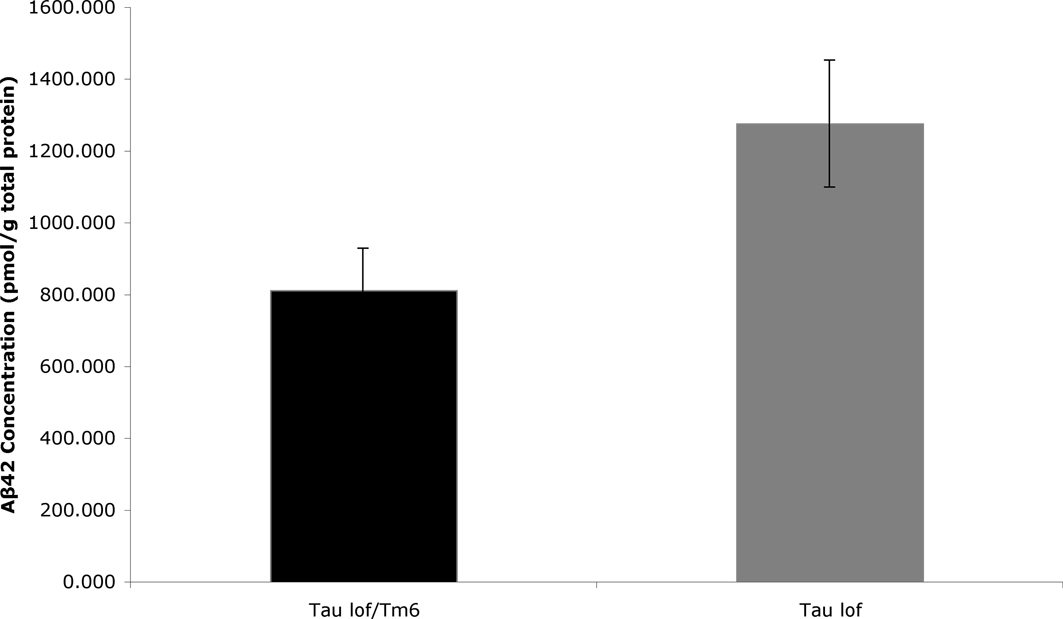


Supplementary figure 5

Supplement: Figure S5 — Reduction in tau levels does not affect amyloid levels of Arctic Aβ42 flies. Protein levels of UAS-ArcAβ42/+;elavGS Tau EP3203/Tau Dfc to UAS-ArcAβ42/+;elavGS Tau EP3203/TM6 flies flies on +RU486 SY medium, were measured by ELISA at 21 days post-induction. Data are presented as the average protein concentration, ± standard error of mean, data were compared using one-way ANOVA and student t-test, number of independent tests (n) = 3. No significant difference was seen in the levels of Aβ42. (0.08 MB DOC) [file pgen.1001087.s005.doc]
