# Supplementary material for: Inhibition of GSK-3 Ameliorates Aβ Pathology in an Adult-Onset Drosophila Model of Alzheimer's Disease
Source: PLoS Genet. 2010 Sep 2;6(9):e1001087. doi: 10.1371/journal.pgen.1001087 (PMC2932684; doi:10.1371/journal.pgen.1001087)
Supplement: Figure S4 — Full-length Tau (P10636), the longest CNS Tau isoform (P10636-8) and Drosophila Tau were aligned by Clustal W alignment. *Shaded in sky and light blue are sites conserved between human and Drosophila tau that are phosphorylated by GSK-3 in vitro. The two Ser sites 262 and 356 (light blue) had available human tau antibodies that detect those conserved sites in Drosophila. ** Shaded in red are likely GSK-3 sites and Abeta sites (212 and 214 for Abeta) that we had available antibodies for and tested in Drosophila, but did not work since they are not conserved. ***Shaded in yellow is an Abeta site (Ser 422) that is conserved in Drosophila, which we had an antibody for; Ser 262 is also a likely Abeta site. (0.03 MB DOC) [file pgen.1001087.s004.doc]

Supplementary Figure 4

CLUSTAL 2.0.12 multiple sequence alignment

sp|P10636|TAU_HUMAN MAEPRQEFEVMEDHAGTYGLGDRKDQGGYTMHQDQEGDTDAGLKESPLQT 50

sp|P10636-8|TAU_HUMAN MAEPRQEFEVMEDHAGTYGLGDRKDQGGYTMHQDQEGDTDAGLKESPLQT 50

tr|Q9VB13|Q9VB13_DROME MADVLEKSSLLDAVP-------------------------------PLGD 19

**: :: .::: . **

sp|P10636|TAU_HUMAN PTEDGSEEPGSETSDAKSTPTAEDVTAPLVDEGAPGKQAAAQPHTEIPEG 100

sp|P10636-8|TAU_HUMAN PTEDGSEEPGSETSDAKSTPTAEDVTAPLVDEGAPGKQAAAQPHTEIPEG 100

tr|Q9VB13|Q9VB13_DROME PHPPLPHQQLQQEAAAAAAANAAPPAPPQQQQPPPHQLQQQQPQQQQLQQ 69

* ..: .: : * ::..* :.* :: .* : **: : :

sp|P10636|TAU_HUMAN TTAEEAGIGDTPSLEDEAAGHVTQEPESGKVVQEGFLREPGPPGLSHQLM 150

sp|P10636-8|TAU_HUMAN TTAEEAGIGDTPSLEDEAAGHVTQ-------------------------- 124

tr|Q9VB13|Q9VB13_DROME KPANARANQDQKEGDNDSG------------------------------- 88

..*: . * . ::::.

sp|P10636|TAU_HUMAN SGMPGAPLLPEGPREATRQPSGTGPEDTEGGRHAPELLKHQLLGDLHQEG 200

sp|P10636-8|TAU_HUMAN --------------------------------------------------

tr|Q9VB13|Q9VB13_DROME --------------------------------------------------

sp|P10636|TAU_HUMAN PPLKGAGGKERPGSKEEVDEDRDVDESSPQDSPPSKASPAQDGRPPQTAA 250

sp|P10636-8|TAU_HUMAN --------------------------------------------------

tr|Q9VB13|Q9VB13_DROME --------------------------------------------------

sp|P10636|TAU_HUMAN REATSIPGFPAEGAIPLPVDFLSKVSTEIPASEPDGPSVGRAKGQDAPLE 300

sp|P10636-8|TAU_HUMAN --------------------------------------------------

tr|Q9VB13|Q9VB13_DROME --------------------------------------------------

sp|P10636|TAU_HUMAN FTFHVEITPNVQKEQAHSEEHLGRAAFPGAPGEGPEARGPSLGEDTKEAD 350

sp|P10636-8|TAU_HUMAN --------------------------------------------------

tr|Q9VB13|Q9VB13_DROME --------------------------------------------------

sp|P10636|TAU_HUMAN LPEPSEKQPAAAPRGKPVSRVPQLKARMVSKSKDGTGSDDKKAKTSTRSS 400

sp|P10636-8|TAU_HUMAN -------------------------ARMVSKSKDGTGSDDKKAK------ 143

tr|Q9VB13|Q9VB13_DROME -------------------------VDESTQEKDRNGP------------ 101

. ::.** .*.

sp|P10636|TAU_HUMAN AKTLKNRPCLSPKLPTPGSSDPLIQPSSPAVCPEPPSSPKHVSSVTSRTG 450

sp|P10636-8|TAU_HUMAN --------------------------------------------------

tr|Q9VB13|Q9VB13_DROME --------------------------------------------------

sp|P10636|TAU_HUMAN SSGAKEMKLKGADGKTKIATPRGAAPPGQKGQANATRIPAKTPPAPKTPP 500

sp|P10636-8|TAU_HUMAN ----------GADGKTKIATPRGAAPPGQKGQANATRIPAKTPPAPKTPP 183

tr|Q9VB13|Q9VB13_DROME ---------------------------------NSPSSPVKTPTSTSSKP 118

*:. *.***.:..: *

sp|P10636|TAU_HUMAN SSGEPPKSGDRSGYSSPGSPGTPGSRSRTPSLPTPPTREPKKVAVVRTPP 550

sp|P10636-8|TAU_HUMAN SSGEPPKSGDRSGYSSPGSPGTPGSRSRTPSLPTPPTREPKKVAVVRTPP 233

tr|Q9VB13|Q9VB13_DROME DKS----GTSRPPSATPSNKSAPKSRSASKNRLLLKTPEPEPVKKVPMN- 163

... . .*. ::*.. .:* *** : . * **: * *

sp|P10636|TAU_HUMAN KSPSSAKSRLQTAPVPMPDLKNVKSKIGSTENLKHQPGGGKVQIINKKLD 600

sp|P10636-8|TAU_HUMAN KSPSSAKSRLQTAPVPMPDLKNVKSKIGSTENLKHQPGGGKVQIINKKLD 283

tr|Q9VB13|Q9VB13_DROME --------KVQVGHAPSPNLKAVRSKIGSLDNATYKPGGGHVKIESKKID 205

::*.. .* *:** *:***** :* .::****:*:* .**:*

sp|P10636|TAU_HUMAN LSNVQSKCGSKDNIKHVPGGGSVQIVYKPVDLSKVTSKCGSLGNIHHKPG 650

sp|P10636-8|TAU_HUMAN LSNVQSKCGSKDNIKHVPGGGSVQIVYKPVDLSKVTSKCGSLGNIHHKPG 333

tr|Q9VB13|Q9VB13_DROME IKAAPRIEAKND--KYMPKGGEKKIVTTKLQWN-AKSKIGSLENAAHKPG 252

:. . ..:* *::* **. :** . :: . ..** *** * ****

sp|P10636|TAU_HUMAN GGQVEVKSEKLDFKDRVQSKIGSLDNITHVPGGGNKKIETHKLTFRENAK 700

sp|P10636-8|TAU_HUMAN GGQVEVKSEKLDFKDRVQSKIGSLDNITHVPGGGNKKIETHKLTFRENAK 383

tr|Q9VB13|Q9VB13_DROME GGDKKIETLKMDFKDKAKPKVGSTANVKHQPGGGDIKIQTQKLEIKAQSK 302

**: :::: *:****:.:.*:** *:.* ****: **:*:** :: ::*

sp|P10636|TAU_HUMAN AKTDHGAEIVYKSPVVSGDTSPRHLSNVSSTGSIDMVD--SPQLATLADE 748

sp|P10636-8|TAU_HUMAN AKTDHGAEIVYKSPVVSGDTSPRHLSNVSSTGSIDMVD--SPQLATLADE 431

tr|Q9VB13|Q9VB13_DROME VGSLDNVKHKPGGGEKKIFDDKDYLKNVEHSVALTTPPTQSPLPSMTASG 352

. : ...: . . . :*.**. : :: ** : *.

sp|P10636|TAU_HUMAN VSASLAKQGL 758

sp|P10636-8|TAU_HUMAN VSASLAKQGL 441

tr|Q9VB13|Q9VB13_DROME ADENLNQQS- 361

.. .* :*.
